# Catalytic Pyrolysis of Post-Consumer Polypropylene over a Regenerated FCC Catalyst Under Moderate Vacuum: Gasoline- and Diesel-Range Fuels with a Low Aromatic Content

**DOI:** 10.3390/polym18212760

**Published:** 2026-11-06

**Authors:** Caterine Donoso, Gabriela Taipe-Andagua, Liliana Guzmán-Beckmann, Alberto Coronas, Alexis Debut, Sebastian Taco-Vasquez, Lucia E. Manangon-Perugachi, Andrés Chico-Proaño, Joan Carles Bruno

**Affiliations:** 1 Universitat Rovira i Virgili, Departamento de Ingeniería Mecánica, Av. Paisos Catalans, 26, 43007 Tarragona, Spain; 2 Departamento de Ciencias de la Energía y Mecánica, Universidad de las Fuerzas Armadas ESPE, Av. General Rumiñahui s/n y Ambato, Sangolquí 171103, Ecuador; jessicataipe21@gmail.com; 3 Centro de Nanociencia y Nanotecnología, Universidad de las Fuerzas Armadas ESPE, Sangolquí 171103, Ecuador; apdebut@espe.edu.ec; 4 Departamento de Ingeniería Química, Facultad de Ingeniería Química y Agroindustria, Escuela Politécnica Nacional EPN, Quito 170143, Ecuador; liliana.guzman@epn.edu.ec (L.G.-B.); sebastian.taco@epn.edu.ec (S.T.-V.); andres.chico@epn.edu.ec (A.C.-P.); 5 Departamento de Metalurgia Extractiva, Facultad de Ingeniería Química y Agroindustria, Escuela Politécnica Nacional EPN, Quito 170143, Ecuador; lucia.manangon@epn.edu.ec

**Keywords:** polypropylene pyrolysis, waste valorisation, FCC catalyst, vacuum pyrolysis, aromatic suppression, TGA kinetics, fuel properties

## Abstract

Post-consumer polypropylene (PP) waste is a growing environmental liability in developing economies with limited recycling infrastructure. This study presents an integrated kinetic-to-reactor approach for the catalytic pyrolysis of three compositionally distinct post-consumer PP streams, combining a moderate vacuum (0.45 bar absolute) with a regenerated fluid catalytic cracking (FCC) catalyst to produce diesel-range fuel while valorising two industrial waste streams. The activation energies derived from thermogravimetric analysis (*β* = 30 °C min^−1^, N_2_) using the Coats–Redfern method were 178.3 kJ mol^−1^ for the near-pure isotactic PP feedstock, consistent with random chain scission, and 209.2 and 351.9 kJ mol^−1^ for the UV-stabilised and PP/HDPE streams, respectively, reflecting the feedstock composition; the DTG profile informed the selection of 450 °C as the reactor set point. At the best condition within the studied domain (Cat/PP = 0.03 g g^−1^, 2 h, 450 °C), the regenerated FCC catalyst achieved 75.10 wt% liquid yield, a 50% improvement over thermal pyrolysis, with a low aromatic content of 1.69 area%,attributed primarily to the moderate acidity of the regenerated catalyst, versus the 10–25 wt% typical of atmospheric zeolitic pyrolysis. Vacuum distillation yielded a diesel fraction that mainly complies with the ASTM D975 Grade 2-D S15 parameters (cetane index 68.92, sulphur 14.1 ppm) without hydrotreatment, demonstrating a technically viable waste-to-fuel pathway where post-consumer polyolefins and refinery FCC by-products are simultaneously abundant.

## 1. Introduction

The accumulation of post-consumer polypropylene (PP) waste represents a mounting environmental liability across Latin American and developing-economy contexts, where collection infrastructure and tertiary recycling capacity remain severely constrained. In Ecuador alone, PP constitutes approximately 11% of the 4.14 million tonnes of solid waste generated annually, yet only a negligible fraction is subject to material or energy recovery [1]. At the regional scale, Latin America generated approximately 28.8 million tonnes of plastic waste in 2020, which was equivalent to 12.4% of total municipal solid waste against a recycling rate of only 4.5%, underscoring both the magnitude of the disposal burden and the largely unrealised potential for waste-to-resource conversion within a circular economy framework [2]. Pyrolysis, the thermochemical decomposition of organic polymers under inert atmosphere, represents a technically viable pathway for tertiary recycling of PP into fuel-grade liquid hydrocarbons, combustible gases, and a carbonaceous solid residue, and has been extensively investigated as a core technology for sustainable waste management and energy recovery from plastic streams [3,4].

Thermogravimetric analysis (TGA) is a fundamental tool for characterising the thermal decomposition behaviour of polymers before scaling to reactor systems. By monitoring mass loss as a function of temperature under controlled conditions, TGA provides characteristic decomposition temperatures and kinetic parameters that inform reactor design, process optimisation, and energy management [5]. Kinetic modelling based on isoconversional or model fitting approaches quantifies the activation energy (*E_a_*) and pre-exponential factor (A) of the Arrhenius rate expression, which govern the reaction rate’s sensitivity to temperature [6]. Among the available methods, the isoconversional approaches of Friedman [7], Kissinger–Akahira–Sunose [8], and Flynn–Wall–Ozawa [9,10] are the most widely applied in polymer kinetics [11], but the ICTAC Kinetics Committee recommends that they be applied across multiple heating rates [6]. Because these isoconversional (model-free) methods require several heating rates, they are not applicable to a single-rate dataset such as that of the present work; a model-fitting method, such as the Coats–Redfern equation, is therefore adopted, as it derives the apparent activation energy from a single thermogram.

Previous kinetic studies of PP pyrolysis have primarily employed virgin polymer [5,12], whereas post-consumer PP waste differs in that it contains thermal and mechanical degradation products, residual additives, colourants, and contaminants from prior service [13]. These compositional variations can shift the apparent activation energy relative to virgin PP values. Aboulkas et al. [14] reported *E_a_* = 179–188 kJ mol^−1^ for virgin PP using the Friedman, KAS, and FWO isoconversional methods, while Westerhout et al. [15] obtained a range of 180–240 kJ mol^−1^ for various PP grades. Quantifying the effect of waste composition on kinetic parameters is therefore of practical importance for process design with heterogeneous feedstocks.

Catalytic pyrolysis of PP with zeolitic catalysts significantly improves liquid yield and selectivity towards fuel range hydrocarbons compared to thermal pyrolysis [16,17]. Fluid catalytic cracking (FCC) catalysts, based on zeolite Y in an aluminosilicate matrix, are well suited for catalytic PP cracking, owing to their controlled Brønsted acidity, defined pore architecture, and availability as spent catalysts from refinery operations [16,17]. The regeneration of spent FCC catalyst by calcination at 700 °C restores a substantial fraction of catalytic activity at low material cost, making it economically attractive for waste to fuel applications [16,18]. Operating pressure remains underexplored relative to temperature and catalyst, yet it significantly affects the rates and product distribution of polyolefin degradation [19]. Elevated pressure favours gas, cycloalkanes, and aromatics [20], whereas moderate vacuum raises the condensable (liquid) yield and suppresses secondary cracking in PP pyrolysis, giving a liquid enriched in diesel-range compounds under purely thermal conditions [21]. On this basis, reduced pressure is expected to lower the effective boiling points of the condensable products and to disfavour secondary aromatisation via Diels–Alder cyclisation, a hypothesis that motivates the present study, in which moderate vacuum is combined with a regenerated FCC catalyst to redirect the product distribution toward lighter, gasoline-range hydrocarbons.

The environmental imperative for advanced plastic waste management has accelerated research into chemical recycling routes, with pyrolysis increasingly recognised as a technically viable and environmentally preferable alternative to landfilling and incineration, particularly for contaminated or mechanically non-recyclable polyolefin streams [22]. Within a circular economy framework, the simultaneous valorisation of two industrial waste streams post-consumer thermoplastics and spent refinery catalysts represents a higher-order sustainability objective beyond single-waste treatment, since it addresses both the uncontrolled accumulation of solid plastic waste and the hazardous waste classification of spent FCC catalyst under refinery operations [23,24]. Recent critical reviews have emphasised that catalytic pyrolysis, when coupled with refinery-derived catalysts and tailored process conditions, constitutes a promising pathway to align plastic waste valorisation with circular economy targets [22,25].

The present work addresses three research gaps: (i) the Coats–Redfern kinetic characterisation of three compositionally distinct post-consumer PP waste streams from Ecuador, with the quantification of composition effects on the apparent activation energy; (ii) the integration of TGA-derived kinetic parameters into the selection of reactor operating conditions for catalytic pyrolysis; and (iii) the first reported compliance assessment of PP-derived pyrolysis diesel with both ASTM D975 Grade 2-D S15 [26] and the Ecuadorian NTE INEN 1489 standard [27] under combined moderate vacuum and regenerated FCC catalyst conditions.

## 2. Materials and Methods

### 2.1. Feedstock Characterisation

Post-consumer PP waste was collected from recycling centres in Ecuador. Three samples of distinct origins were prepared: M1 (mixed PP articles chairs, bottle caps, containers with visual evidence of HDPE contamination), M2 (PP roofing panels, relatively homogeneous composition), and M3 (commercial transparent PP pellets containing UV-stabiliser additives, of the type routinely incorporated into polypropylene for outdoor service to retard UV-induced degradation [28]). All samples were washed with 2 wt% NaOH solution, rinsed with deionised water, dried at 80 °C for 48 h, and granulated to approximately 1 mm particle size.

The PP waste was characterised by Fourier Transform Infrared Spectroscopy (FTIR) using a Perkin-Elmer Spectrum One spectrometer in transmittance mode (4000–400 cm^−1^, resolution 4 cm^−1^, 32 scans) following ASTM E1252-98 [29]. Strong absorption bands at 1164.61, 973.28, and 841 cm^−1^ confirmed isotactic PP. Elemental analysis (C, N, S) was performed at the Laboratorio del Departamento de Petróleos, Energía y Contaminación (DPEC), Universidad Central del Ecuador, using an Elementar Vario Macro Cube analyser operated in combustion (Dumas) mode. Hydrogen was obtained by difference, since the instrumental hydrogen reading was not consistent with the sample mass balance (footnote to Table 1). Higher heating values (HHVs, gross calorific values) were measured at the same laboratory by bomb calorimetry on a Parr oxygen-bomb calorimeter (Parr Instrument Company, Moline, IL, USA) (in-house procedure PNE/DPEC/P/M002, ref. ASTM D240) [30] at 543.1 mmHg and 19.5 °C, without correction for sulphur or moisture content. Differential scanning calorimetry (DSC) was conducted on a Netzsch DSC 204 F1 (Netzsch-Gerätebau GmbH, Selb, Germany) at 10 °C min^−1^ under N_2_ according to ASTM D3418-12 [31] to determine melting temperature (Tm).

### 2.2. Thermogravimetric Analysis (TGA)

Thermogravimetric experiments were conducted on a TGA 1 STAR System (Mettler-Toledo, Greifensee, Switzerland;)model B448304771). Samples of 11–15 mg were placed in alumina crucibles and heated from ambient temperature to 600 °C at a constant heating rate of *β* = 30 °C min^−1^ in a dynamic nitrogen atmosphere (flow rate 30 mL min^−1^) to prevent oxidative reactions. The TG and derivative thermogravimetric (DTG) curves were recorded continuously as functions of temperature. Characteristic decomposition temperatures were extracted: T5%, the temperature at 5% mass loss (onset temperature); T50%, the temperature at 50% conversion; *T_max_*, the temperature at maximum decomposition rate (DTG peak); and T95%, the temperature at 95% conversion. Conversion (*α*) was defined as shown in Equation (1), where m_0_, m, and m_∞_ are the initial, instantaneous, and final residual masses, respectively.

(1) $$\propto =\frac{\left(m_{0}-m\right)}{\left(m_{0}-m_{\infty }\right)}$$

### 2.3. Kinetic Modelling Framework

The general rate expression for non-isothermal solid-state decomposition at a constant heating rate is given by Equation (2):

(2) $$\frac{d\propto }{dT}=\frac{A}{\beta }\;e^{-\frac{E_{a}}{RT}}\;f(\propto )$$

where *f*(*α*) is the differential reaction model, A is the pre-exponential factor (s^−1^), *E_a_* is the activation energy (kJ mol^−1^), and *R* is the universal gas constant (8.314 J mol^−1^ K^−1^). Its integration yields Equation (3):

(3) $$g\left(\alpha \right)=\int_{0}^{\alpha }\frac{d\alpha }{f\left(\alpha \right)}=\frac{A}{\beta }\int_{T_{0}}^{T}exp\left(-\frac{E_{a}}{RT}\right)dT$$

A first-order reaction model was assumed throughout, i.e., *f*(*α*) = (1 − *α*) and *g*(*α*) = −ln(1 − *α*), consistent with the single-peak DTG profiles observed and with the random chain scission mechanism reported for PP pyrolysis in the literature [14,15].

### 2.4. Kinetic Model Fitting

The Coats–Redfern method was applied to the TGA data at *β* = 30 °C min^−1^ for each of the three PP samples. This model-fitting method derives the kinetic parameters from a single thermogram [32] and is therefore appropriate for the single heating rate used in this work.

Based on the Coats–Redfern approximation of the temperature integral [33], the linearised form is given by Equation (4), where *g*(*α*) is the integral form of the reaction model. For a first-order reaction, *g*(*α*) = −ln(1 − *α*), so that a plot of ln[−ln(1 − *α*)/*T*^2^] against 1/*T* yields a straight line of slope −*E_a_*/*R* and intercept ln[*AR*/(*βE_a_*)] [32].

(4) $$ln\left(\frac{g\left(\alpha \right)}{T^{2}}\right)=ln\left(\frac{AR}{E_{a}\cdot \beta }\right)-\frac{E_{a}}{RT}$$

Kinetic parameters (*E_a_* and *A*) were obtained from the slope and intercept of the corresponding linearisation.

### 2.5. FCC Catalyst Regeneration and Characterisation

Spent FCC catalyst was obtained from an Ecuadorian state-owned refinery. The regeneration process was conducted in two stages following the protocol established by Palmay et al. (2023) [34], who applied the same regeneration sequence to a different PP feedstock under atmospheric pressure. The present work extends this approach by operating under a moderate vacuum (0.45 bar), a pressure regime not investigated in [34], and by employing three compositionally distinct post-consumer PP waste streams characterised by TGA-derived kinetics, constituting a novel process-integrated contribution absent from the prior study. In the first stage (chemical treatment), ethanol was used to remove sulphides and sulphur oxides adsorbed in the catalyst structure. Solutions of 200 mL ethanol were prepared and stirred at 300 rpm for 14 h with the spent catalyst, maintaining a ratio of 10 mL solvent per gram of catalyst. The resulting solution was filtered, and the solid product was dried at 120 °C for 2 h to ensure the complete evaporation of the solvent. This chemical treatment was essential for removing sulphur compounds that would otherwise interfere with catalytic activity and poison active sites. In the second stage (thermal treatment), the chemically treated catalyst underwent controlled calcination using a three-step temperature programme (ramp 2): (1) heating from ambient temperature to 350 °C at 50 °C/h and maintaining at 350 °C for 1 h; (2) heating to 450 °C at 25 °C/h and maintaining at 450 °C for 1.5 h; and then (3) heating to 700 °C at 50 °C/h and maintaining at 700 °C for 2 h. The total thermal treatment time was approximately 22.5 h. After calcination, samples were cooled gradually to ambient temperature. The high-temperature treatment (700 °C) was necessary for complete gasification of deposited coke into CO_2_ and SO_2_, though it also caused some structural modifications, including sintering and pore reorganisation. Alternative regeneration methods, such as bioleaching, have also been reported [35].

The catalyst was characterised by X-ray diffraction (XRD; Empyrean diffractometer (Malvern Panalytical, Almelo, The Netherlands), Cu Kα radiation, λ = 1.5406 Å, 10–70° 2θ) and scanning electron microscopy with energy-dispersive X-ray spectroscopy (SEM/EDS; TESCAN MIRA3 (TESCAN, Brno, Czech Republic)) at the Centro de Nanociencia y Nanotecnología (CENCINAT), Universidad de las Fuerzas Armadas ESPE, Sangolquí, Ecuador. N_2_ adsorption–desorption isotherms at 77 K (BET surface area and total pore volume; Micromeritics AutoChem II 2920 (Micromeritics Instrument Corp., Norcross, GA, USA)) and temperature-programmed desorption of NH_3_ (NH_3_-TPD) for total acidity quantification (5 °C min^−1^, TCD detection; the operating conditions are in Table S8 and the desorption profile is in Figure S5) were performed at the Universidad Central del Ecuador, Quito, Ecuador. Crystalline phases were quantified by Rietveld refinement using HighScore Plus (Malvern Panalytical, Almelo, The Netherlands).

### 2.6. Catalytic Pyrolysis Experiments

Catalytic pyrolysis was conducted in a 1 litre borosilicate glass batch reactor equipped with a QCC SCADA-based temperature control system. Approximately 320 g of PP waste (M2 composition) was loaded per run with the regenerated FCC catalyst. The reactor was evacuated to an absolute pressure of 0.45 bar (moderate vacuum) and heated from ambient temperature to 450 °C at 9 °C min^−1^. This operating temperature was selected based on the *T_max_* of sample M2 (450 °C) determined by TGA, ensuring full conversion at a minimum thermal input. A 3 × 3 factorial experimental design was applied, varying the catalyst-to-PP mass ratio (Cat/PP = 0.02, 0.03, 0.04 g g^−1^) and residence time at a set temperature (1, 1.5, 2 h). Products were collected as condensed liquid, permanent gas (measured by gas counter), and solid residue (weighed post-experiment). A thermal pyrolysis reference experiment (MT, no catalyst, 2.0 h) was also performed. All pyrolysis experiments were performed in triplicate; liquid yields are reported as mean values on a mass basis, with a maximum relative standard deviation (RSD) of 2.9% across replicate runs.

### 2.7. Product Characterisation

The liquid products were characterised by GC–MS using a PerkinElmer Clarus 680 gas chromatograph coupled to a Clarus SQ 8 T mass spectrometer (PerkinElmer, Waltham, MA, USA), fitted with a Zebron ZB-5MS capillary column (Phenomenex, Torrance, CA, USA; 30 m × 0.25 mm × 0.25 μm). Helium was used as carrier gas at 1 mL min^−1^, the injector and detector were held at 250 °C, and the injection volume was 0.5 μL. The oven programme was: 50 °C (3 min), ramp at 7 °C min^−1^ to 220 °C, then 25 °C min^−1^ to 250 °C (6.51 min hold), for a total run time of 35 min. Compound identification was performed with TurboMass software (PerkinElmer, Waltham, MA, USA) by NIST 2011 library matching [36] (minimum similarity 80%) of the 60 most representative peaks. No internal standard or compound-specific response factors were used; the reported compositions therefore correspond to relative chromatographic peak-area percentages and are semiquantitative. The liquid fraction from the best condition run (T6) was separated by vacuum distillation into a gasoline fraction (IBP–200 °C) and a diesel fraction (200–350 °C). Both fractions were characterised by the ASTM test methods described in Section 3.8, with specifications compared against ASTM D975 Grade 2-D S15 (diesel) and NTE INEN 935 Extra [37] (gasoline).

The gaseous fraction was captured in Tedlar bags and analysed on the same PerkinElmer Clarus 680 gas chromatograph (PerkinElmer, Waltham, MA, USA) according to ASTM D1945-03 (2014) [38] and ASTM D3588-98 (2017) [39]. Individual gas components (C1-C6 hydrocarbons, CO_2_) were quantified, and the gas properties including relative density, average molecular weight, density, higher and lower heating values, and compressibility factor, were calculated. The composition and properties of both thermal and catalytic pyrolysis gases were compared to assess the influence of the FCC catalyst on product distribution. Hydrogen was not quantified; as a light permanent gas, it is not resolved by this hydrocarbon-oriented column configuration and gives a low, non-linear response with a thermal-conductivity detector and helium carrier gas [40], and it is prone to permeation loss during storage in Tedlar/PVF sampling bags [41].

## 3. Results

### 3.1. Feedstock Characterisation Results

Table 1 presents the elemental composition and heating values of the three PP waste samples alongside literature data for virgin PP [12,14]; the same data, together with the statistical comparison of HHVs against virgin PP, are reported in Table S1. All three samples are dominated by carbon and hydrogen, confirming their suitability as hydrocarbon fuel precursors. The carbon contents of 81.4–85.7 wt% and hydrogen contents of 14.0–16.9 wt% are consistent with predominantly polyolefinic composition. The nitrogen content is low (≤1.500 wt%), and the sulphur contents range from 0.155 to 0.320 wt%. Comparable sulphur levels (0.1 wt%) have been reported for waste PP and attributed to processing additives and residues of the natural gas from which propylene is obtained [42]. The distribution of this sulphur among the pyrolysis products is examined in Section 3.8.

The elevated nitrogen content of M3 (1.500 wt%) is attributed to UV stabilisers (specifically 2,4-di-tert-butyl-6-(5-chloro-2H-benzotriazol-2-yl)phenol, identified by FTIR) present in the commercial pellet formulation. The molar H/C ratios of the three feedstocks, 2.13 (M1), 1.95 (M2), and 2.47 (M3), bracket the value of 2.0 expected for a polyolefin backbone, with M2 being the closest to it, in agreement with its identification as the purest iPP sample. The HHVs of the three feedstocks (44.14–45.27 MJ kg^−1^, mean 44.68 ± 0.57 MJ kg^−1^) do not differ significantly from the 44 MJ kg^−1^ reported for virgin PP (one-sample *t*-test, *p* = 0.172; Table S1), indicating that the additive and contaminant loads of the post-consumer streams do not measurably reduce their energy content. FTIR spectra of all three samples (Figure S1) confirmed the characteristic bands of isotactic PP (iPP): CH_3_ asymmetric stretch (2950 cm^−1^), CH_2_ deformation (1455 cm^−1^), CH_3_ symmetric deformation (1375 cm^−1^), C–C skeletal band (1165 cm^−1^), the helical conformation band common to crystalline and amorphous phases used as PP reference (973 cm^−1^), and the helical regularity band of iPP (841 cm^−1^) [43]. DSC analysis of M2 confirmed a melting temperature of Tm = 167.8 ± 1.1 °C, characteristic of iPP.

### 3.2. Thermogravimetric Analysis

Figure 1 illustrates the thermal decomposition profiles of the three PP waste samples at *β* = 30 °C min^−1^ under N_2_ (full-scale TG and DTG curves are given in Figure S2). All samples exhibit a single prominent DTG peak, confirming that pyrolysis proceeds predominantly as a single step process. This is consistent with the homolytic C–C bond cleavage and random chain scission mechanism characteristic of polyolefin thermal degradation, and with prior literature for PP [12,14]. The onset of significant mass loss (T5%) occurred at approximately 400 °C, with maximum decomposition rates (*T_max_*) at 473 °C (M1), 450 °C (M2), and 478 °C (M3). Complete conversion (T95%) was achieved by approximately 500 °C for all samples.

Table 2 summarises the characteristic temperatures derived from TG-DTG analysis; the corresponding maximum conversions and the shift in *T_max_* relative to neat PP are reported in Table S3. The onset temperatures (T5%) range from 368 °C (M2) to 398 °C (M3), and the maximum degradation temperatures (*T_max_*) from 450 °C (M2) to 478 °C (M3). These values are consistent with the literature’s ranges of 400–500 °C for PP pyrolysis [4,15]. The relatively lower *T_max_* of M2 compared to M1 and M3 reflects its predominantly iPP composition and comparatively low additive loading; as the closest approximation to a pure PP matrix in this study, M2 undergoes uninhibited random chain scission at the characteristic temperature range reported for neat isotactic PP [4,15]. The higher *T_max_* of M3 (478 °C) coincides with the presence of the UV benzotriazole stabiliser (2,4-di-tert-butyl-6-(5-chloro-2H-benzotriazol-2-yl)phenol) identified by FTIR in this feedstock.

The variation in maximum degradation temperatures among the three samples is consistent with their compositional differences as characterised by FTIR (spectra in Figure S1; band assignments in Table S2). Sample M2 (*T_max_* = 450 °C), the closest approximation to a pure iPP matrix in this study, degraded at the lowest temperature, in agreement with the characteristic decomposition range reported for neat isotactic PP [4,15]. These literature values provide a quantitative frame for the present results: Buekens [44] reported a *T_max_* of 425 °C for PP, over a decomposition range of 340–460 °C, against 475 °C for HDPE, over 340–500 °C. The three *T_max_* values measured here (450, 473, and 478 °C) therefore fall between these two reference endpoints, and their ordering tracks the polyethylene content of each feedstock rather than any single functional group. Its FTIR spectrum exhibits a single CH2 rocking absorption at 725 cm^−1^ and the iPP diagnostic bands at 973 and 841 cm^−1^, with no evidence of HDPE contamination. FTIR absorptions at 556 and 472 cm^−1^ in the M2 spectrum are attributable to Fe–O stretching vibrations of iron oxide pigment (Fe_2_O_3_), consistent with the characteristic red colouration of the roofing panels; iron oxide has been reported to act as a mild co-catalyst for PP thermal degradation through radical-mediated mechanisms [45,46], which may marginally contribute to the lower *T_max_* of M2, though the dominant factor is its high iPP purity and absence of thermal stabilisers. Sample M1 (*T_max_* = 473 °C) contains HDPE in addition to PP, confirmed by CH2 stretching bands at 2902 and 2849 cm^−1^ and a CH2 rocking band shifted to 731 cm^−1^, superimposed on the iPP diagnostic bands at 972 and 841 cm^−1^. Polyethylene chains consist exclusively of secondary C–H bonds (–CH2–), which require a higher activation energy for homolytic cleavage than the tertiary C–H bonds (–CH(CH3)–) present in every PP repeat unit [25,47], raising the composite DTG peak above that of pure M2. Its *T_max_* of 473 °C is, in fact, nearly coincident with the 475 °C reported for neat HDPE [44], indicating that the polyethylene fraction governs the position of the DTG peak in this sample. Sample M3 exhibited the highest *T_max_* (478 °C) among the three samples. The FTIR spectrum of M3 shows absorptions at 3297 cm^−1^ (intramolecular O–H···N hydrogen bond of the benzotriazole chelate), 3075 cm^−1^ (aromatic C–H), 1551 cm^−1^ (triazole ring C=N), and 1264 cm^−1^ (C–N stretch), directly confirming the presence of the UV stabiliser (2,4-di-tert-butyl-6-(5-chloro-2H-benzotriazol-2-yl)phenol) in the feedstock. The band at 3297 cm^−1^ is assigned to the intramolecular hydrogen-bonded O–H (enol form) of the 2-(2-hydroxyphenyl)benzotriazole chromophore; a broad hydroxyl band near 3346 cm^−1^ has been reported for structurally analogous chloro-substituted hydroxyphenyl-benzotriazoles in the solid state [48]. The presence of this stabiliser is consistent with the highest *T_max_* observed among the three samples. Weak, sharp features between 3600 and 3760 cm^−1^ were detected in the three spectra (Figure S1) and were attributed to uncompensated atmospheric water vapour rather than to hydroxyl groups of the samples; they were therefore excluded from the structural assignment.

### 3.3. Kinetic Model Analysis

#### 3.3.1. Direct Linearisation Parameters

Figure 2 presents the Coats–Redfern first-order linearization of the three PP samples, obtained by plotting ln[−ln(1 − *α*)/*T*^2^] against 1/T for the single non-isothermal experiment at *β* = 30 °C min^−1^; the corresponding regression equations, activation energies, and pre-exponential factors are compiled in Table S4. All three regressions yielded R^2^ ≥ 0.989. Although a high coefficient of determination confirms internal linearity, it is well established that R^2^ alone is an insufficient criterion for kinetic model discrimination, as multiple mechanistic models can yield comparably high R^2^ values for the same dataset [6,11]. The Coats–Redfern model-fitting approach adopted here yields the apparent activation energy from a single thermogram [32,49] and is therefore appropriate for the compositionally heterogeneous feedstocks studied.

A striking observation is the markedly higher activation energy of M1 (351.9 kJ mol^−1^) compared to M2 (178.3 kJ mol^−1^) and M3 (209.2 kJ mol^−1^). This is attributed to the HDPE fraction of M1, as HDPE decomposes with a substantially higher activation energy than PP, with reported values of 238–247 kJ mol^−1^ for HDPE against 179–188 kJ mol^−1^ for PP [14], and 262 kJ mol^−1^ for HDPE against 190 kJ mol^−1^ for PP [44]. Fitting the bimodal PP/HDPE decomposition of M1 as a single first-order step yields a lumped, effective activation energy that exceeds that of either pure component, consistent with the FTIR evidence of mixed polyolefin content in this sample.

The activation energy of M2 (178.3 kJ mol^−1^) is consistent with the values reported for pure PP—179–188 kJ mol^−1^ [14], 190 kJ mol^−1^ [44], and 183.7 kJ mol^−1^ [50]—and is comparable to the 152 kJ mol^−1^ obtained by the Coats–Redfern first-order model for PP, containing plastic mixtures [49]. This confirms M2 as the most representative pure PP sample in this study. The elevated activation energy of M3 (209.2 kJ mol^−1^) relative to M2 is consistent with the presence of the UV-stabiliser additive package identified by FTIR, which raises the apparent energy barrier for the onset of decomposition.

#### 3.3.2. Coats–Redfern Kinetic Parameters

The first-order Coats–Redfern method yielded apparent activation energies of 351.9, 178.3, and 209.2 kJ mol^−1^ for M1, M2, and M3, respectively. The high value obtained for M1 is attributed to its mixed PP and HDPE composition, because HDPE generally exhibits a higher thermal-degradation energy barrier than PP [14,44]. When the decomposition of both polymers is represented by a single global reaction, the resulting value should be interpreted as an effective lumped parameter rather than as an intrinsic kinetic constant.

Sample M2, identified as the most homogeneous isotactic PP feedstock, exhibited the lowest apparent activation energy, 178.3 kJ mol^−1^. This value agrees with activation energies previously reported for PP pyrolysis using Coats–Redfern and other kinetic approaches [14,32,44,49,50]. The higher value obtained for M3 may be associated with the UV-stabiliser and additive package identified by FTIR in this feedstock.

Because the experiments were conducted at a single heating rate, these values correspond to global model-fitting parameters and must not be interpreted as activation energy profiles as a function of conversion [6,11].

#### 3.3.3. Model Adequacy

The Coats–Redfern regressions showed satisfactory linearity, with R^2^ values between 0.9893 and 0.9974, supporting the use of a first-order global model to describe the principal decomposition stage. However, a high coefficient of determination does not demonstrate that the selected model represents a unique reaction mechanism, because different solid-state kinetic models may provide similarly satisfactory fits to the same thermogravimetric dataset [6,11,51].

Accordingly, the activation energies and pre-exponential factors obtained in this study should be interpreted as apparent kinetic parameters dependent on the assumed reaction model, the selected conversion interval, and the experimental conditions [33,51]. Their principal purpose is to compare the thermal behaviour of the three post-consumer PP feedstocks and support the selection of the reactor operating temperature.

#### 3.3.4. Mechanistic Interpretation and Reactor Design Implications

The predominantly single DTG peak and the satisfactory Coats–Redfern linearisation indicate that the main mass-loss interval can be represented by a global first-order reaction. For M2, the apparent activation energy of 178.3 kJ mol^−1^ is consistent with values reported for the thermal degradation of isotactic PP and supports random chain scission as the predominant overall decomposition pathway [4,14,15,44,50]. Nevertheless, the Coats–Redfern method alone cannot establish an elementary reaction mechanism, and the interpretation must also consider the DTG profile, feedstock composition, and available literature evidence [6,11,51].

The maximum decomposition-rate temperature of M2, T_max_ = 450 °C, was used as a reference for selecting the reactor set point. This temperature lies within the range commonly reported for the catalytic pyrolysis of PP using FCC-derived catalysts [16]. However, direct equivalence between thermogravimetric conversion and batch-reactor conversion cannot be assumed, as the two systems differ in heating rate, sample mass, pressure, heat transfer, mass transfer, and catalyst contact. Therefore, 450 °C should be regarded as an experimentally supported reactor set point rather than as a kinetically demonstrated best operating temperature. Under the factorial conditions evaluated, the highest liquid fraction yield was obtained at 450 °C, a catalyst-to-PP mass ratio of 0.03 g g^−1^, and a residence time of 2 h.

### 3.4. Regenerated FCC Catalyst Characterisation

Table 3 compares the physicochemical properties of the regenerated FCC catalyst with fresh and regenerated/equilibrium FCC catalysts reported in the literature [52,53]. The BET surface area of 134.1 m^2^ g^−1^ and total pore volume of 0.067 cm^3^ g^−1^, lower than the values reported for fresh FCC catalyst (158.13 m^2^ g^−1^, 0.147 cm^3^ g^−1^) [52], reflect the combined effect of two simultaneous deactivation mechanisms: reversible pore occlusion by residual carbonaceous deposits accumulated during the cracking cycle, and irreversible hydrothermal dealumination and sintering of the aluminosilicate matrix induced by exposure to 700 °C in the presence of steam generated during coke combustion. XRD analysis confirmed the partial preservation of the crystalline zeolite Y framework (71.3%), consistent with structural degradation driven primarily by high-temperature hydrothermal conditions. Despite these losses, the regenerated catalyst retains sufficient textural and structural integrity to remain catalytically functional.

The total acidity of the regenerated catalyst measured by NH_3_-TPD (66.1 μmol NH_3_ g^−1^; Figure S5) is markedly lower than the combined Brønsted and Lewis acidity reported for fresh FCC catalyst by pyridine-FTIR (210 μmol Py g^−1^ at 150 °C) [52]; although the two techniques are not directly comparable, both point to the substantial loss of acid sites that accompanies FCC service and regeneration [52]. Despite this reduction, the Brønsted acid sites retained in the regenerated catalyst are sufficient to initiate carbenium-ion cracking of PP chains at 450 °C. The bulk Si/Al molar ratio of the regenerated catalyst (0.93) lies between the values derived for the Brazilian equilibrium catalysts (0.86–0.89) and for fresh commercial FCC catalysts (1.2–2.0) characterised by Souza et al. [53], while framework-level dealumination of the zeolite phase is evidenced independently by the unit-cell parameter a_0_ = 24.256 Å, characteristic of strongly dealuminated (ultra stable) Y zeolite with framework Si/Al ≥ 6 [54]. Despite the reduction in surface area and acidity relative to fresh catalyst, the regenerated FCC catalyst represents a compelling economic case; it is recovered from a refinery by-product stream at low acquisition cost, in contrast to commercial fresh zeolite-based cracking catalysts.

These characterisation results collectively confirm that the regeneration protocol (calcination at 700 °C for 2 h in static air) partially restores catalytic functionality without full recovery of fresh catalyst properties. The diffraction pattern and the Rietveld phase quantification of the regenerated catalyst are provided in Figure S3. The residual NH_3_-TPD acidity (66.1 μmol NH_3_ g^−1^) is nevertheless sufficient for practical application; Kongngoen et al. [16] demonstrated that a minimum Brønsted acidity of approximately 50 μmol NH_3_ g^−1^ is sufficient to initiate β-scission cracking of polyolefin chains at temperatures above 400 °C, a threshold comfortably exceeded by the regenerated catalyst used in this work (66.1 μmol NH_3_ g^−1^). The mean particle size of 44.7 μm, below the 50–150 μm range typical of spray-dried FCC microspheres [54], is attributed to attrition and mechanical fragmentation of catalyst particles during FCC operation, which generates a significant proportion of fines (approximately 42%) and reduces the mean diameter of the recovered functional fraction. The substantial montmorillonite content quantified by XRD (26.5%) suggests thermal restructuring of the clay matrix, which likely contributes to a pore volume reduction without significantly affecting the zeolite Y cracking activity responsible for liquid yield improvement. The abundant fines and inter-particle voids observed in the SEM micrographs are consistent with the generation of macro-defects by attrition.

Energy-dispersive X-ray spectroscopy (EDS) of the regenerated catalyst yielded Si and Al as the dominant elements (21.01 and 21.68 wt% as measured), corresponding to normalised oxide contents of 45.62 wt% SiO_2_ and 41.58 wt% Al_2_O_3_ (Table 3; the elemental composition is listed in Table S5 and the corresponding spectra in Figure S4). This alumina-rich bulk composition is closer to that of the regenerated atmospheric-residue FCC catalyst characterised by Zakariyaou et al. (39.04 wt% SiO_2_, 50.28 wt% Al_2_O_3_) [52] than to the silica-rich fresh commercial formulations reported by Souza et al. (56.2–65.3 wt% SiO_2_) [53], consistent with an equilibrium catalyst in which successive reaction–regeneration cycles have restructured the aluminosilicate matrix.

Iron is the most significant contaminant detected: 3.33 wt% as measured (4.84 wt% as Fe_2_O_3_ on the normalised oxide basis), roughly one order of magnitude above the fresh FCC catalysts (0.27–0.82 wt% Fe) and substantially above the equilibrium catalysts (0.36–0.90 wt% Fe) characterised by Souza et al. [53], reflecting cumulative feedstock-derived deposition over successive cracking cycles combined with residual structural iron from the clay matrix [53]. Iron deposits accumulate preferentially near the outer surface of FCC particles, blocking pores and restricting molecular diffusion [53,55]. This surface restriction is consistent with the lower surface area (134.1 m^2^ g^−1^) and total pore volume (0.067 cm^3^ g^−1^) of the regenerated catalyst relative to the fresh FCC catalyst characterised in [52] (158.13 m^2^ g^−1^, 0.147 cm^3^ g^−1^; Table 3).

Lanthanum (2.64 wt% as measured; 3.14 wt% as La_2_O_3_ on the normalised oxide basis) confirms rare-earth exchange of the zeolite, in line with the La-exchanged faujasite phase identified by XRD and with the RE_2_O_3_ contents of 2.16–3.65 wt% reported for Brazilian equilibrium catalysts [53]. Rare-earth cations limit the extent of framework dealumination under FCC conditions, stabilising the aluminosilicate lattice [56]; La retention after calcination at 700 °C is consistent with the preservation of measurable NH_3_-TPD acidity (66.1 µmol NH_3_ g^−1^) and zeolite Y crystallinity (71.3%), confirming the functional viability of the regenerated catalyst for PP cracking. Sodium remains low (0.14 wt%; 0.19 wt% as Na_2_O), within the range of fresh commercial catalysts (0.19–0.42 wt% Na_2_O) [53], while the Mg enrichment (2.19 wt%) and trace Zn (0.03 wt%) suggest minor contamination from refinery process streams rather than the original catalyst formulation.

Scanning electron microscopy of the regenerated FCC catalyst (Figure 3) reveals morphological features consistent with the combined effects of thermal regeneration and cumulative mechanical attrition during prior FCC service. At a low magnification (BSE detector, 500 μm scale bar), the particle population exhibits a heterogeneous size distribution with a bimodal character comprising larger spherical particles (estimated 30–60 μm) and an abundant fraction of sub 20 μm fines occupying the interstitial spaces directly consistent with the measured mean particle diameter of 44.7 μm and the particle recovery analysis indicating approximately 42% fines generated by attrition. This polydisperse distribution is a well-documented consequence of particle–particle and particle–wall collisions in the riser and cyclone sections of FCC units, where mechanical fragmentation progressively reduces the mean diameter of the circulating inventory; indeed, the main causes of catalyst deactivation in service include the attrition factor associated with fluid dynamic stresses and thermal cycling, in addition to coke deposition on active sites and pore blockage [18]. At a higher magnification (50 μm scale bar), the spherical morphology characteristic of spray-dried FCC catalyst bodies formed from aqueous slurries of zeolite Y, binder, and clay filler by the spray-drying process into 50–150 μm spheres [53,54,57,58] is clearly preserved in the larger particles, confirming that the calcination regeneration protocol did not collapse the global particle architecture. However, the particle surfaces appear rough and heterogeneous under BSE imaging, with adherent sub-micron debris and irregular surface features consistent with partial surface ablation and residual mineral deposits; this rough surface texture is characteristic of FCC catalysts that have undergone cracking cycles and has been attributed to adhesion of reaction products and structural modifications occurring at the particle surface during operation [53,54,57,58]. The inter-particle void space visible between the larger spheres at high magnification is qualitatively consistent with the generation of macro-defects and interparticle voids from attrition rather than an increase in intrinsic micropore volume, a distinction supported by the low BET surface area (134.1 m^2^ g^−1^) and total pore volume (0.067 cm^3^ g^−1^) relative to fresh FCC catalyst (158.13 m^2^ g^−1^, 0.147 cm^3^ g^−1^) [52]. This is consistent with the established understanding that heterogeneity in the spatial porosity distribution at multiple length scales governs the physicochemical and catalytic properties of FCC particles, with pore space geometry playing a determining role in mass transport toward the active zeolite domains [54]. Collectively, the SEM micrographs confirm that the regenerated catalyst retains the spherical morphology required for stable fluidisation while exhibiting the textural modifications, such as surface roughening, fines generation, and inter-particle void enlargement, that are characteristic of industrial-grade equilibrium FCC catalysts after thermal and mechanical cycling.

### 3.5. Catalytic Pyrolysis: Product Distribution

Figure 4 presents the product mass balances for all nine experimental runs and the thermal reference (MT). Seven of the nine catalytic runs yielded higher liquid fractions than the thermal baseline (50.00 wt%), confirming the promoting role of regenerated FCC catalyst on liquid yield under moderate vacuum conditions. The two exceptions—T4 (41.24 wt%) and T7 (31.84 wt%.%)—correspond to the shortest residence time (1.0 h) combined with elevated catalyst loadings (0.03 and 0.04 g g^−1^), a result discussed in detail below. The best condition within the domain (T6: Cat/PP = 0.03 g g^−1^, 2 h) yielded 75.10 wt% liquid, 6.37 wt% gas, and 18.53 wt% solid residue, representing a 50% improvement over the thermal reference, in line with liquid yields reported for integrated plastic pyrolysis processes [59].

Examination of the factorial design trends in Figure 4 reveals that residence time exerts the strongest influence on liquid yield within the ranges tested. Increasing the residence time from 1.0 to 2.0 h at Cat/PP = 0.02 g g^−1^ raised the liquid yield from 51.12 to 63.25 wt% (+23.7%), accompanied by a reduction in solid residue from 36.90 to 25.23 wt%. A more pronounced improvement is observed at Cat/PP = 0.03 g g^−1^, where the liquid fraction increased from 41.24 to 75.10 wt% as the residence time was extended from 1.0 to 2.0 h, while the solid residue fell from 50.60 to 18.53 wt%. This behaviour is consistent with the kinetically controlled nature of the cracking reactions at 450 °C: the Brønsted acid sites on the regenerated FCC catalyst initiate β-scission of the molten polymer chains, but sufficient contact time is required to achieve high conversion of high-molecular-weight oligomers and wax intermediates into condensable liquid-range hydrocarbons [16,60].

The effect of catalyst loading is nonmonotonic and strongly dependent on residence time. At 1.0 h, increasing Cat/PP from 0.02 to 0.04 g g^−1^ produced a monotonic decrease in the liquid yield (T1: 51.12 wt%, T4: 41.24 wt%, T7: 31.84 wt%), with the highest solid residue observed at T7 (62.32 wt%). This behaviour at short contact times is attributed to mass transfer limitations inherent to batch reactors processing molten polymers: the polymer melt forms a viscous film over the catalyst particles, limiting diffusion of cracked volatiles and resulting in incomplete polymer-to-product conversion [53]. At 1.5 and 2.0 h, the catalyst loading effect exhibits a clear maximum at Cat/PP = 0.03 g g^−1^, with liquid yields peaking at 68.52 wt% (T5) and 75.10 wt% (T6), then declining to 53.59 wt% (T8) and 57.95 wt% (T9) at Cat/PP = 0.04 g g^−1^. The reduction in liquid yield at the highest catalyst loading and longer residence times is attributed to the onset of secondary cracking, whereby liquid-range products undergo further acid-catalysed fragmentation into permanent gases, thereby reducing the net condensable fraction [17,61]. This finding has direct implications for process scale-up; Cat/PP = 0.03 g g^−1^ represents the best balance between catalytic activity and selectivity toward liquid products, minimising both catalyst consumption and over-cracking losses.

Comparing the best condition catalytic run (T6) with the thermal reference (MT) highlights the role of the regenerated FCC catalyst beyond a simple yield increase. The thermal run produced 50.00 wt% liquid and a substantial 35.00 wt% gas fraction, while T6 achieved 75.10 wt% liquid with only 6.37 wt% gas a 5.5-fold reduction in gas yield. The suppression of gaseous product formation under catalytic conditions reflects the ability of zeolitic Brønsted acid sites to redirect cracking selectivity toward liquid-range C_5_–C_12_ products through controlled β-scission rather than the unselective radical chain scission mechanism that governs thermal pyrolysis [16]. Furthermore, the catalytic liquid fraction showed a markedly higher gasoline selectivity (70.7%) compared to the thermal reference (53.8%), confirming the role of the regenerated FCC catalyst in directing product distribution toward lighter, higher-value fractions.

#### Statistical Evaluation of the Factorial Design and Response Surface

The effect of the operating factors on the liquid-fraction yield was evaluated by a two-way ANOVA with replication (triplicate runs; 18 degrees of freedom of pure error) and a second-order response surface (RSM) fitted to the 27 observations. Residence time was the dominant factor (F = 56.0, *p* < 0.0001), followed by the catalyst-to-PP ratio (F = 17.7, *p* = 0.0001) and their interaction (F = 6.6, *p* = 0.0019); all three effects were statistically significant (α = 0.05). The quadratic model (coded variables X_1_ = Cat/PP, X_2_ = time) was Y = 64.77 + 12.02·X_2_ − 3.88·X_1_ − 9.94·X_1_^2^ − 4.72·X_2_^2^ + 3.49·X_1_X_2_ (R^2^ = 0.80; adj. R^2^ = 0.75; p_model < 0.0001), with a significant linear time term (*p* < 0.0001) and a significant curvature in Cat/PP (X_1_^2^, *p* = 0.0015), indicating an interior maximum in the catalyst-to-PP ratio (≈0.03 g g^−1^) and a monotonic increase in yield with time over the range studied. The surface (Figure S6) locates the best condition within the domain at Cat/PP = 0.03 g g^−1^ and 2.0 h (treatment T6), with a predicted liquid yield of 72 wt%, in agreement with the experimental value of T6 (75.1 wt%). The stationary point of the model is a maximum located just outside the domain (≈2.1 h), suggesting that slightly longer residence times could marginally increase the liquid yield.

As an integrity caveat, the response surface showed a statistically significant lack of fit (F = 6.8, *p* = 0.003) and the experimental variability between replicates was appreciable (SD ≈ 5 percentage points); the quadratic model is therefore used as a descriptive, exploratory tool to locate the best condition within the domain, and not as a validated quantitative predictor. The significance of the factors rests on the replicated ANOVA, not on the fitted surface.

### 3.6. Liquid Product Composition (GC-MS Analysis)

Table 4 presents the GC–MS composition of the T6 (best condition catalytic) and MT (thermal reference) liquid fractions, grouped by the carbon number range. The characteristic PP pyrolysis marker 2,4-dimethyl-1-heptene (C_9_H_18_, propylene trimer) was the base peak in both runs (Tables S6 and S7), confirming that chain scission governs the primary fragmentation mechanism under both conditions [62,63]. The T6 liquid contained 54.10 area % total aliphatics, dominated by alkenes (32.16 area %) with a significant cycloalkane contribution (12.43 area %), while the aromatic content (1.69 area %) remained an order of magnitude below the ≈24.6 area % total aromatics (5.13 area % monoaromatics plus 19.44 area % naphthalenes) reported for catalytic PP pyrolysis over spent FCC catalyst [64], as discussed in Section 4. An estimate of the oil elemental composition derived from the GC–MS speciation, following the peak-area/brute-formula method of Nardella et al. [65] (Table S9), yields C ≈ 84.7 wt%, H ≈ 14.1–14.2 wt% and O ≤ 1.2 wt% for both liquids (H/C ≈ 2.0, O/C ≤ 0.011), matching the polypropylene backbone and confirming a predominantly hydrocarbon, essentially oxygen-free oil, in contrast to the oxygen-rich oils obtained in biomass–plastic co-pyrolysis.

The carbon number distribution reveals the difference in cracking depth between catalytic and thermal conditions. The C_9_ fraction dominates both runs (T6: 23.28 area %; MT: 25.35 area %), with 2,4-dimethyl-1-heptene as the base peak in both chromatograms [62,63]. The most notable diagnostic difference is the complete absence of C_20_+ compounds in T6 (0 area %) versus 16.43 area % in MT, where cyclotetracosane (C_24_H_48_) dominates, providing direct chromatographic evidence that the regenerated FCC catalyst achieves a high degree of cracking of the high-molecular-weight wax intermediates, here defined as the hydrocarbon fraction above C_20_ [66], that persist under purely thermal conditions [18]. The light C_4_–C_8_ fraction is correspondingly higher in T6 (18.22 area %) than in MT (10.88 area %), and aromatics remained low in both runs (T6: 1.69 area %; MT: 0.94 area %). The unidentified fraction in T6 (38.66 area %) exceeds that of MT (20.46 area %) and is concentrated in 14 unresolved peaks in the C_10_–C_13_ range (of 60 representative peaks, Tables S6 and S7), consistent with a complex mixture of branched constitutional isomers beyond NIST library matching capacity [62]. Oxygenated species, absent from the PP backbone, are attributed to contaminants inherent to the recycled feedstock (food-contact residues and processing additives) [46,59,67]: MT contained cyclic alcohols, a C_22_ diester, and a propyl vinyl ether (3.76 wt% ethers), whereas the only oxygenates in T6 were the short-chain aldehydes 2-methylpentanal and heptanal.

### 3.7. Gaseous Products Composition and Properties

The gaseous fraction, while representing a smaller mass percentage of products compared to liquids, constitutes a valuable co-product with potential applications as fuel gas or chemical feedstock. Table 5 presents the GC composition of the gas fractions from the thermal reference run (MT) and the best condition catalytic run T6.

Propane was the dominant hydrocarbon in both thermal (43.59 wt%) and catalytic T6 (46.25 wt%) gases, consistent with the C_3_-selective chain scission of the isotactic PP repeat unit [25]. The C_4_–C_5_ range showed pronounced redistribution under catalytic conditions: isobutane increased from 0.22 to 3.67 wt% and n-butane from 6.85 to 16.44 wt%, while isopentane decreased from 24.49 to 1.94 wt% and n-pentane increased from 1.63 to 14.47 wt%, a pattern consistent with carbenium-ion isomerisation and β-scission over the Brønsted sites of the zeolite Y phase [17,61]. These shifts increased the LPG-range fraction (C_3_ + C_4_) from 50.66 to 66.36 wt% and reduced the C_5_+ fraction from 28.04 to 16.82 wt%, redirecting the gas toward lower-molecular-weight, higher-value fractions [16,17].

CO_2_ decreased from 10.06 wt% (thermal) to 4.69 wt% (catalytic), and water was 0.01 wt% in MT versus 0.36 wt% in T6. Both observations trace back to the oxygenated contaminants of the recycled feedstock identified in Section 3.6 [46,67]: the lower CO_2_ reflects the reduced oxygenate content of the T6 liquid and the shorter vapour-phase residence time under 0.45 bar, while the higher water content is consistent with acid-catalysed dehydration of cyclic alcohol intermediates over the Brønsted sites [45].

Hydrogen was not quantified in this work. Although H_2_ is a primary gaseous product of PP pyrolysis [68], its formation by dehydrogenation is minor at 450 °C and becomes significant only at substantially higher temperatures [69]; a low H_2_ level is therefore expected under the present conditions. Its absence from the reported composition is attributed to the sampling and detection limitations noted in Section 2.7 (permeation loss in Tedlar/PVF bags and the low, non-linear H_2_ response of the thermal-conductivity detector with helium carrier gas) rather than to a true absence.

The physical and energetic properties of the catalytic T6 gas are summarised in Figure 5. The average molecular weight of 41.20 g mol^−1^ and density of 1.74 g L^−1^ are consistent with the C_3_–C_5_-dominated composition, and the compressibility factor of 1.0000 confirms near-ideal behaviour. The higher and lower heating values (70,677 and 65,088 kJ m^−3^) place the gas between natural gas (≈37,000 kJ m^−3^) and pure propane (≈93,200 kJ m^−3^) [60], the difference from propane reflecting dilution by ethane (8.96 wt%) and CO_2_ (4.69 wt%). This energy density makes the gas suitable as fuel for process heating or as supplementary fuel for the pyrolysis reactor itself, improving the overall energy balance.

### 3.8. Fuel Property Characterisation

Table 6 summarises the ASTM test methods and corresponding NTE INEN norms applied in this work, as well as the fuel properties of the diesel and gasoline fractions recovered from vacuum distillation of the T6 liquid, with comparison against ASTM D975 Grade 2-D S15 and NTE INEN 1489 (diesel) and NTE INEN 935 Extra (gasoline) specification limits.

The diesel fraction (28 vol% of T6 liquid) exhibits a calculated cetane index of 68.92, substantially exceeding both the ASTM D975 minimum of 40 and the NTE INEN 1489 minimum of 45, thereby classifying this product unambiguously as Grade 2-D S15 diesel. This exceptionally high cetane index reflects the high α-olefin and n-alkane contents of the diesel fraction; long-chain linear hydrocarbons are known to exhibit cetane numbers well above 60, combined with the virtual absence of aromatic compounds, which are the principal cetane-depressing components in conventional petroleum diesel [60]. The sulphur content of 14.1 ppm (0.00141 wt%) satisfies the ASTM D975 S15 requirement (≤15 ppm) and the NTE INEN 1489 limit for any commercial diesel grade marketed in Ecuador, without requiring hydrotreatment [18]. The sulphur contents of the gasoline (14.8 ppm) and diesel (14.1 ppm) fractions are equivalent, as both are recovered by vacuum distillation from the same pyrolysis liquid and together account for 97 wt% of it (12 wt% condensate and 85 wt% distillate). Relative to the 1830 ppm of the M2 feedstock, the liquid phase thus retains 0.6% of the sulphur fed to the reactor. The balance is expected in the gas and solid streams, in which volatile sulphur is released as H_2_S while the remainder is retained by the carbonaceous residue, which acts as a heteroatom adsorbent [70].

Kinematic viscosity (2.565 mm^2^ s^−1^ at 40 °C), flash point (87 °C), ash (0.00056 wt%), water and sediment (0.001 vol%), and Conradson carbon residue (0.044 wt%) all comply with both ASTM D975 and NTE INEN 1489 specifications. The diesel distillation curve also satisfies ASTM D975 Grade 2-D limits: T_10_ = 220.2 °C, T_50_ = 249.3 °C, T_90_ = 296.2 °C, and final boiling point = 300.1 °C, all within the normative envelope (T_90_ ≤ 338 °C). The density (0.7813 g cm^−3^) lies below the NTE INEN 1489 range of 0.82–0.85 g cm^−3^ specified for commercial diesel in Ecuador, reflecting the high alkane/α-olefin contents and low aromatic fraction of the pyrolysis diesel; ASTM D975 does not impose a density limit, and this sub-specification density is addressed in the discussion below.

The gasoline fraction (70.7 vol% of T6 liquid) has a research octane number (RON) of 85, below the NTE INEN 935 Extra minimum of 87, and is therefore better suited as a blending component than as a neat fuel. Two gasoline distillation parameters (T_10_ = 97.9 °C, T_50_ = 144.7 °C) exceed NTE INEN 935 limits (≤70 °C and ≤121 °C respectively), attributable to the relatively heavy composition of the vacuum-distilled fraction T_90_ (180.9 °C ≤ 190 °C) and the final boiling point (206.4 °C ≤ 220 °C).

Figure 6 presents bar chart comparisons of key diesel and gasoline properties against ASTM D975/NTE INEN 1489 and NTE INEN 935 specification limits. The bold bar borders indicate compliance with the applicable normative specification.

The two fuel properties that fall outside normative limits warrant contextual discussion. The diesel density (0.7813 g cm^−3^) lies below the NTE INEN 1489 commercial range of 0.82–0.85 g cm^−3^, a direct consequence of the high α-olefin and n-alkane contents of the vacuum-distilled fraction combined with the near-absence of dense aromatic species; notably, ASTM D975 imposes no density specification, confirming this fraction qualifies as Grade 2-D S15 under international standards in the fuel-quality parameters of this study.

In practice, the sub-specification density by NTE INEN 1489 does not preclude fuel use: blending with a conventional diesel fraction at an approximate 40:60 (pyrolysis:fossil) ratio would restore the density to specification range while maintaining the high cetane advantage. The gasoline RON of 85, below the NTE INEN 935 Extra threshold of 87, is lower than the RON of 96 reported by Kassargy et al. [47] for the gasoline-like fraction obtained from the catalytic pyrolysis of PP over fresh USY zeolite at 450 °C, a difference consistent with the moderate acidity of the regenerated FCC catalyst relative to fresh zeolite; it positions the fraction as a blending component requiring octane adjustment rather than a neat fuel. The high HHV of the diesel fraction (46,285 kJ kg^−1^) exceeds that of conventional petroleum diesel (approximately 42,000 kJ kg^−1^) by approximately 10%, attributable to the high hydrogen to carbon ratio of the α-olefin rich fraction and the absence of heteroatom dilution [60]. The anomalously low HHV of the gasoline fraction (31,817 kJ kg^−1^) relative to the diesel fraction and to fossil reference values (approximately 43,500 kJ kg^−1^) is consistent with the elevated oxygenated compound content detected in the catalytic liquid (Table 5; Tables S6 and S7), which dilute the volumetric energy density; this is a direct consequence of the recycled PP feedstock contamination discussed in Section 3.6 [46,67]. Collectively, the fuel characterisation data demonstrate that PP waste pyrolysis under moderate vacuum conditions with regenerated FCC catalyst produces a diesel fraction that is commercially competitive without hydrotreatment, a cost and process simplification advantage of substantial practical relevance for small- to medium-scale waste valorisation in Ecuador and comparable developing economy contexts.

## 4. Discussion

The apparent activation energy of the near-pure isotactic PP feedstock M2 (178.3 kJ mol^−1^) falls at the lower edge of the range reported for PP pyrolysis (179–188 kJ mol^−1^ [14]; 180–240 kJ mol^−1^ across PP grades [15]), consistent with the presence of pre-degraded chains, residual additives and service-life contaminants in post-consumer feedstocks, which lower the apparent energy barrier for chain initiation relative to virgin polymer [13]. It must be emphasised that these Coats–Redfern parameters, obtained from a single heating rate, are global model-fitting parameters and do not constitute an isoconversional (model-free) analysis, which the ICTAC Kinetics Committee recommends be performed only across multiple heating rates [6,11]; they should therefore be interpreted as comparative design inputs rather than mechanism-resolving kinetic constants, and multi-heating-rate experiments remain a priority for their consolidation.

The Coats–Redfern results illustrate the practical risk of reporting kinetic parameters for recycled feedstocks without prior compositional screening: HDPE contamination in M1 raised the apparent activation energy to 351.9 kJ mol^−1^, and the benzotriazole UV stabiliser in M3 raised it to 209.2 kJ mol^−1^, whereas the near-pure iPP sample M2 (178.3 kJ mol^−1^) agreed closely with the virgin PP literature values [14,44,50]. The combination of FTIR fingerprinting and TGA therefore provides a rapid, low-cost screening protocol for heterogeneous polyolefin waste prior to reactor operation, an aspect of direct practical relevance for recycling facilities in developing economies where feedstock provenance is rarely controlled.

At reactor scale, the central finding is that the regenerated FCC catalyst governs the product distribution at the single moderate-vacuum condition studied (0.45 bar). The catalyst governs conversion depth and selectivity, raising liquid yield from 50.00 to 75.10 wt%, eliminating the C_20_+ wax fraction that persists under purely thermal conditions (16.43 wt%), and redistributing the gas phase toward LPG-range products; the low aromatic content (1.69 wt%) is attributed primarily to the moderate acidity of the regenerated catalyst rather than to the reduced pressure, and remains well below the levels typically reported for atmospheric catalytic pyrolysis of polyolefins over zeolitic catalysts (Section 3.6). This decoupling of cracking selectivity from aromatic formation differentiates the present process window from conventional catalytic configurations: Kassargy et al. [47], operating a comparable batch system at 450 °C with fresh USY zeolite at atmospheric pressure, obtained a similar liquid yield for PP (82 wt%) and a gasoline-like fraction with a RON of 96, but a calculated cetane number of 52 for the diesel-like fraction, below the cetane index of 68.92 obtained here; spent FCC upgrading routes, such as those of Kongngoen et al. [16], address wax conversion without targeting aromatic suppression. The nonmonotonic effect of catalyst loading, with over-cracking to permanent gases at Cat/PP = 0.04 g g^−1^ and long residence times, is consistent with acid-site-mediated secondary β-scission [17,61], and identifies Cat/PP = 0.03 g g^−1^ as the ratio giving the best liquid selectivity within the studied domain.

The fuel quality outcome carries the main techno-economic implication of this work. Compliance of the diesel fraction with the ASTM D975 Grade 2-D S15 fuel-quality parameters evaluated, without hydrotreatment, including 14.1 ppm sulphur and a cetane index of 68.92, stems directly from its paraffinic/olefinic composition and low aromatic content, due to the catalyst’s moderate acidity, and removes the single most capital-intensive upgrading step of conventional pyrolysis-to-fuel schemes. Organosulphur species in plastic pyrolysis oils occur predominantly as thiophenes, benzothiophenes, and dibenzothiophenes [70]; suppressing the formation of heavy aromatics therefore also removes the structural vehicle through which sulphur would otherwise be retained in the liquid phase, with the sulphur being partitioned instead towards the gas and solid streams [70]. The two sub-specification parameters (diesel density of 0.7813 g cm^−3^ against NTE INEN 1489, and gasoline RON of 85 against NTE INEN 935) are both blending-correctable and traceable to the same compositional origin: the near-absence of dense, high-octane aromatic species. There is thus an intrinsic trade-off between aromatic suppression and neat-fuel specification compliance, which future pressure-mapping studies across the 0.3–0.6 bar range [71] could resolve by identifying the operating pressure at which aromatic content, density, and octane rating are simultaneously optimised.

The role of vapour-phase history identified here is reinforced by recent fixed-bed studies published in this journal. Papuga et al. demonstrated, through response-surface modelling of waste PP pyrolysis at 450–500 °C, that reactor occupancy dominates liquid yield over both temperature and reaction time, because local overheating of the vapour region above the melt promotes over-cracking of volatiles into non-condensable gases [72]. The moderate vacuum applied in the present work acts directly on this vapour-phase bottleneck, as by shortening the residence time in the hot vapour zone, it suppresses both over-cracking to gas and the sequential Diels–Alder cyclisation–dehydrogenation pathway to aromatics, which explains the simultaneous attainment of a high liquid yield (75.10 wt%) and a low aromatic content (1.69 area %) at a comparatively low set point of 450 °C.

The gas-phase redistribution provides independent mechanistic confirmation of carbenium-ion chemistry on the regenerated catalyst; the seventeen-fold increase in isobutane, the collapse of thermally generated isopentane, and the growth of linear C_4_–C_5_ products (Section 3.7) are signatures of skeletal isomerisation and β-scission over Brønsted acid sites rather than purely radical chain reactions [17,61]. Together with the acid-catalysed cyclisation evidenced by the cycloalkane content of the liquid and the dehydration of feedstock-derived alcohols (Section 3.6 and Section 3.7), these observations confirm that despite the reduced acidity quantified in Section 3.4, the regenerated FCC catalyst retains highly functional Brønsted-site chemistry at the reaction temperature.

The feedstock perspective adopted here, three compositionally distinct post-consumer streams rather than virgin polymer, aligns with recent work on real waste streams. Fulgencio-Medrano et al. showed that pyrolysis oils obtained at 460 °C from streams rejected by the recycling industry can be assimilated into existing refinery units, provided that pre-treatment removes contaminants such as PVC, PET, metals, and cellulosic materials [73]. The NaOH washing and the compositional screening by FTIR and DSC applied to M1–M3 in the present work fulfil the same function at laboratory scale, and the compliance of the resulting diesel fraction with ASTM D975 indicates that the regenerated FCC route can deliver a drop in fuel quality without the additional hydroprocessing stage that refinery integration would otherwise require.

The principal limitations of this study are the single sub-atmospheric pressure point evaluated; the single heating rate used for the kinetic analysis; the batch configuration, whose melt-phase mass-transfer limitations were evident at short residence times [53]; and the absence of multi-cycle regeneration data for the catalyst. Continuous-flow operation, repeated regeneration–reaction cycling, and blending protocols under Ecuadorian fuel market conditions constitute the immediate research agenda, together with the valorisation of the propane-rich gas fraction via catalytic steam reforming toward hydrogen [74], currently under investigation by the authors.

## 5. Conclusions

The Coats–Redfern model-fitting method applied to the thermogravimetric data of the three post-consumer PP feedstocks yielded apparent activation energies of 351.9, 178.3, and 209.2 kJ mol^−1^ for M1, M2, and M3, respectively. The value obtained for the near-pure sample (M2) is consistent with the random chain scission of isotactic PP, whereas the deviations observed for M1 and M3 reflect the influence of HDPE contamination and of UV-stabiliser additives, respectively, confirming that feedstock characterisation is a necessary prior step when reporting kinetic parameters for recycled polyolefin streams. Because a single heating rate was used, these values are global model-fitting parameters rather than isoconversional E_a_(*α*) profiles; multiple heating-rate experiments will be required to obtain a proper isoconversional description. The maximum degradation temperature of 450 °C determined for the reference sample (M2) was adopted as the reactor set point for the subsequent pyrolysis experiments.

Pyrolysis over the regenerated FCC catalyst under moderate vacuum (0.45 bar absolute) produced a liquid-rich product distribution with low aromatic content: at the best condition within the studied domain (Cat/PP = 0.03 g g^−1^, 2 h, 450 °C, 0.45 bar), the liquid yield reached 75.10 wt% with an aromatic content of 1.69 area %. The regenerated catalyst retained approximately 40% of the Brønsted acidity of the fresh material (66.1 μmol NH_3_ g^−1^), a level of acidity consistent with the observed shift in cracking selectivity toward products in the diesel and gasoline boiling ranges. Because the catalytic runs were carried out at a single sub-atmospheric pressure (0.45 bar), the specific contribution of vacuum to this product distribution could not be isolated; given that pressure effects on plastic pyrolysis have received comparatively little attention relative to temperature and catalyst variables [71], a systematic pressure-mapping study across the intermediate vacuum regime (0.3–0.6 bar) is proposed to quantify this effect.

Vacuum distillation of the pyrolysis liquid obtained under this condition (T6) yielded a diesel fraction that complied, without any hydrotreatment step, with the studied fuel-quality parameters of ASTM D975 Grade 2-D S15 and NTE INEN 1489 except for density: a calculated cetane index of 68.92 (ASTM D976), a sulphur content of 14.1 ppm (S15 limit ≤ 15 ppm), a kinematic viscosity of 2.565 mm^2^ s^−1^ at 40 °C, a flash point of 87 °C, a Conradson carbon residue of 0.044 wt%, and a complete T_10_/T_50_/T_90_ distillation envelope within specification. The density of the diesel fraction (0.7813 g cm^−3^) fell below the 0.82–0.85 g cm^−3^ range of NTE INEN 1489 and can be corrected by blending with conventional diesel at an approximate 40:60 pyrolysis-to-fossil ratio. The gasoline fraction showed a research octane number of 85, below the NTE INEN 935 Extra minimum of 87, which is attributable to the moderate acidity of the regenerated catalyst relative to fresh zeolite Y, and is correctable by fractional redistillation or octane-boosting blending. Avoiding the hydrotreatment stage is of practical interest for small- and medium-scale plastic waste valorisation in Ecuador and comparable contexts, where post-consumer polyolefin waste and FCC refinery by-products are simultaneously available.

Future work should address continuous-flow reactor operation, the stability of the catalyst over repeated regeneration cycles, and blending protocols for density correction under Ecuadorian fuel-market conditions. In addition, the C_3_–C_5_-rich gas fraction, dominated by propane (46.25 wt%) and enriched in light olefins under the catalytic conditions studied, constitutes a candidate feedstock for ex situ catalytic steam reforming over supported nickel catalysts (Ni/SiO_2_–Al_2_O_3_) for hydrogen-enriched syngas production [74], a route currently under investigation by the authors.

## Figures and Tables

**Figure 1:**
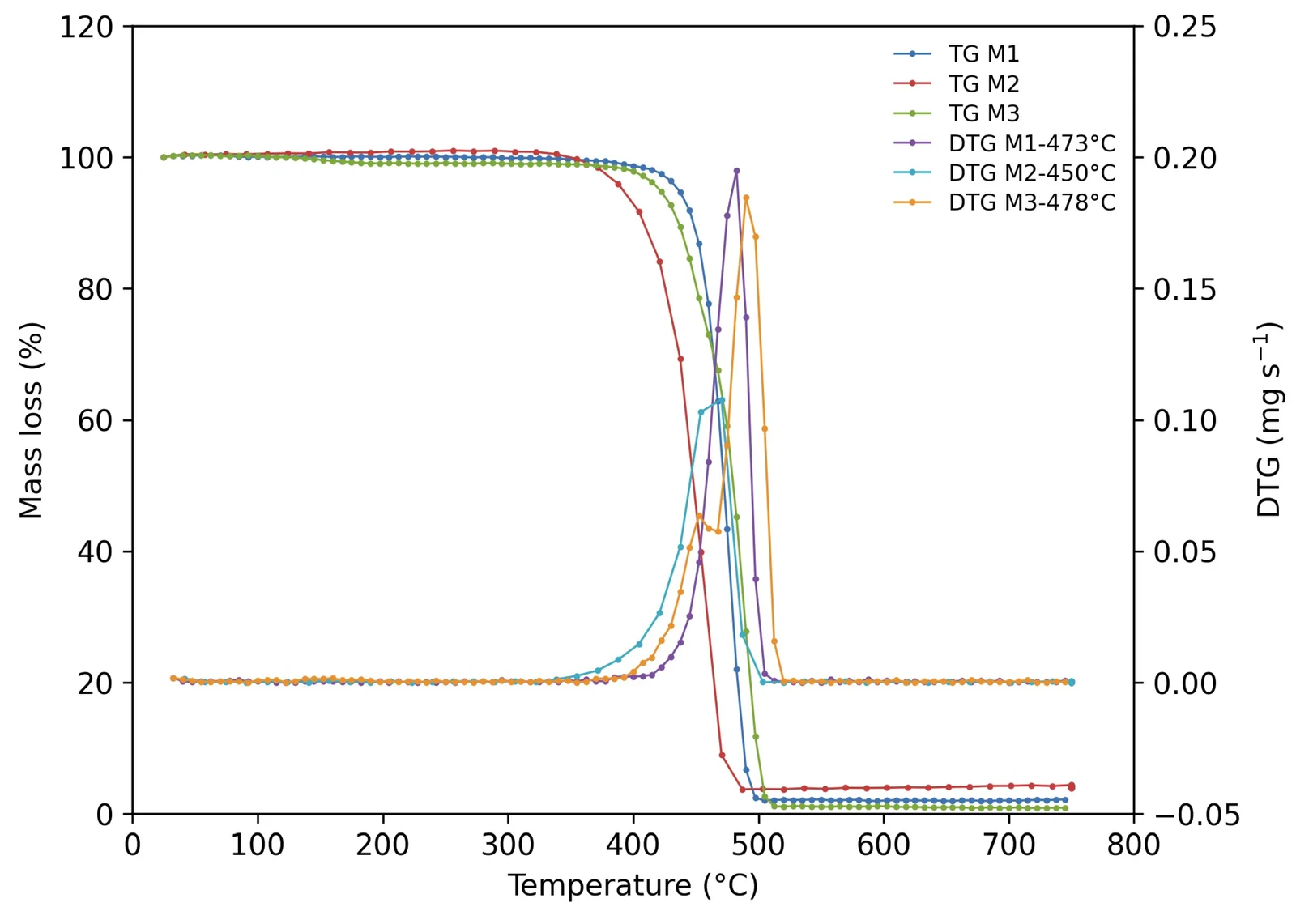
TG and DTG curves of three post-consumer PP waste samples (M1, M2, M3) at *β* = 30 °C min^−1^ under N_2_ atmosphere.

**Figure 2:**
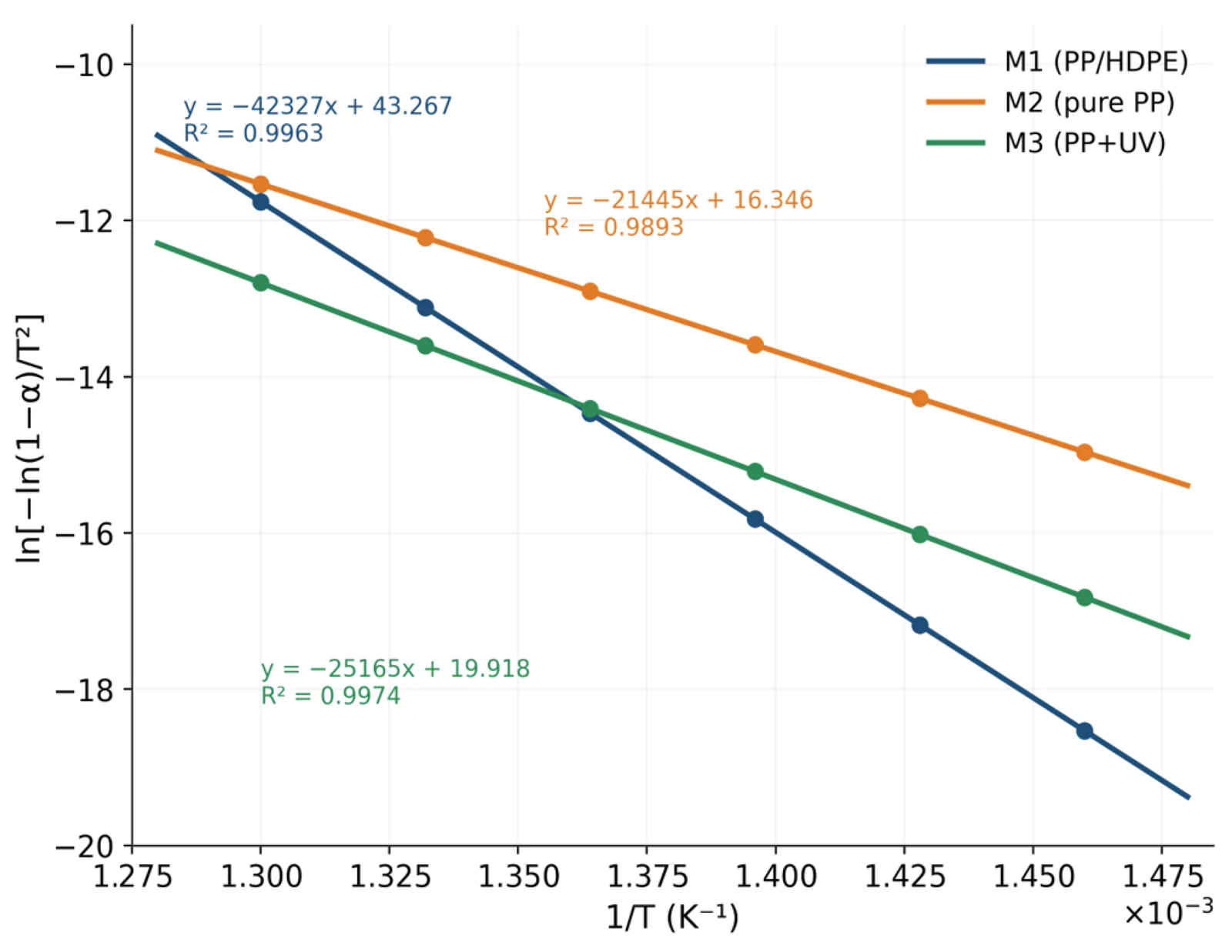
Coats–Redfern first-order linearisation, ln[−ln(1 − *α*)/*T*^2^] versus 1/*T*, for the three PP samples M1, M2, and M3 at *β* = 30 °C min^−1^ (N_2_).

**Figure 3:**
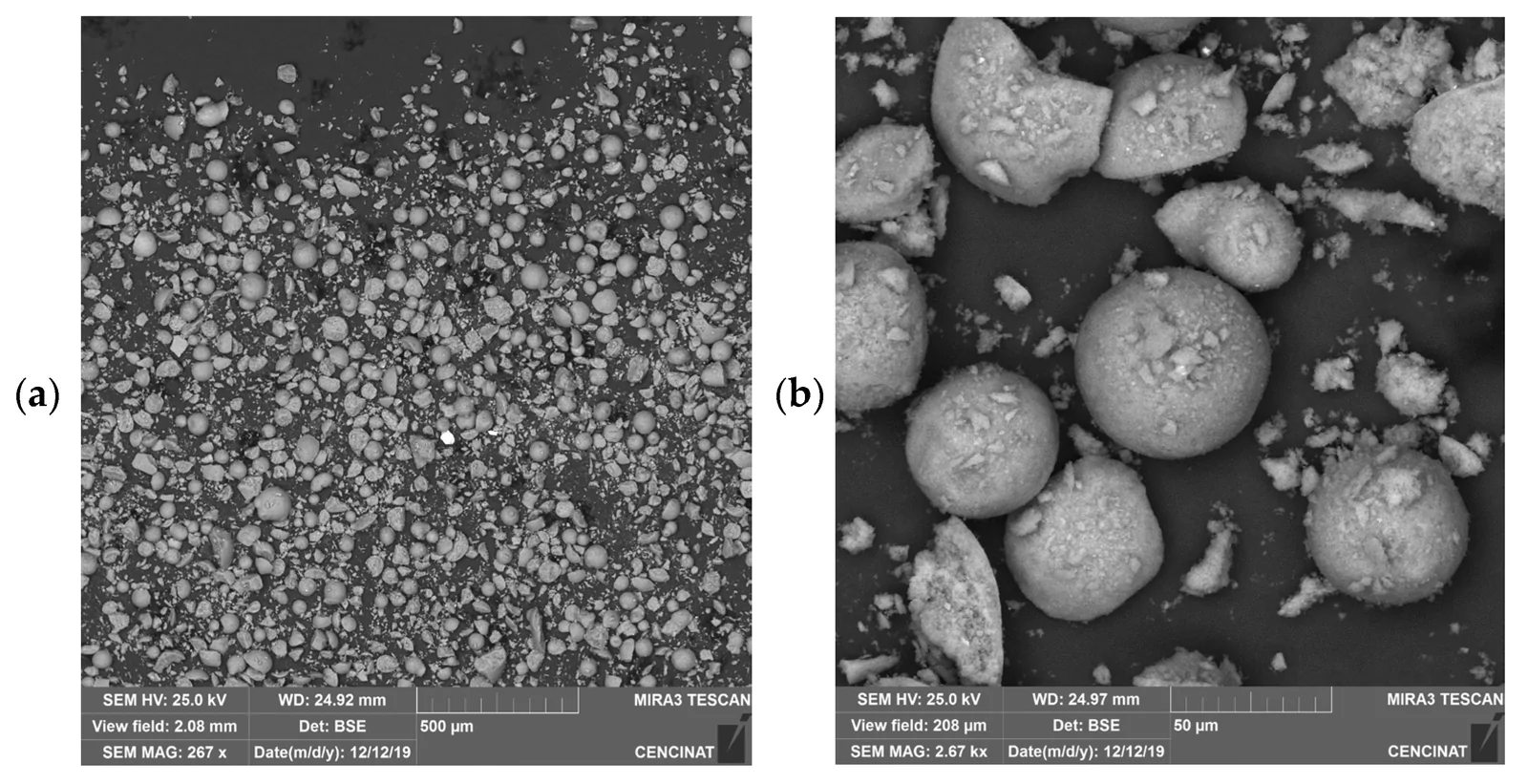
SEM micrographs of the regenerated FCC catalyst at (**a**) 500 μm and (**b**) 50 μm magnification.

**Figure 4:**
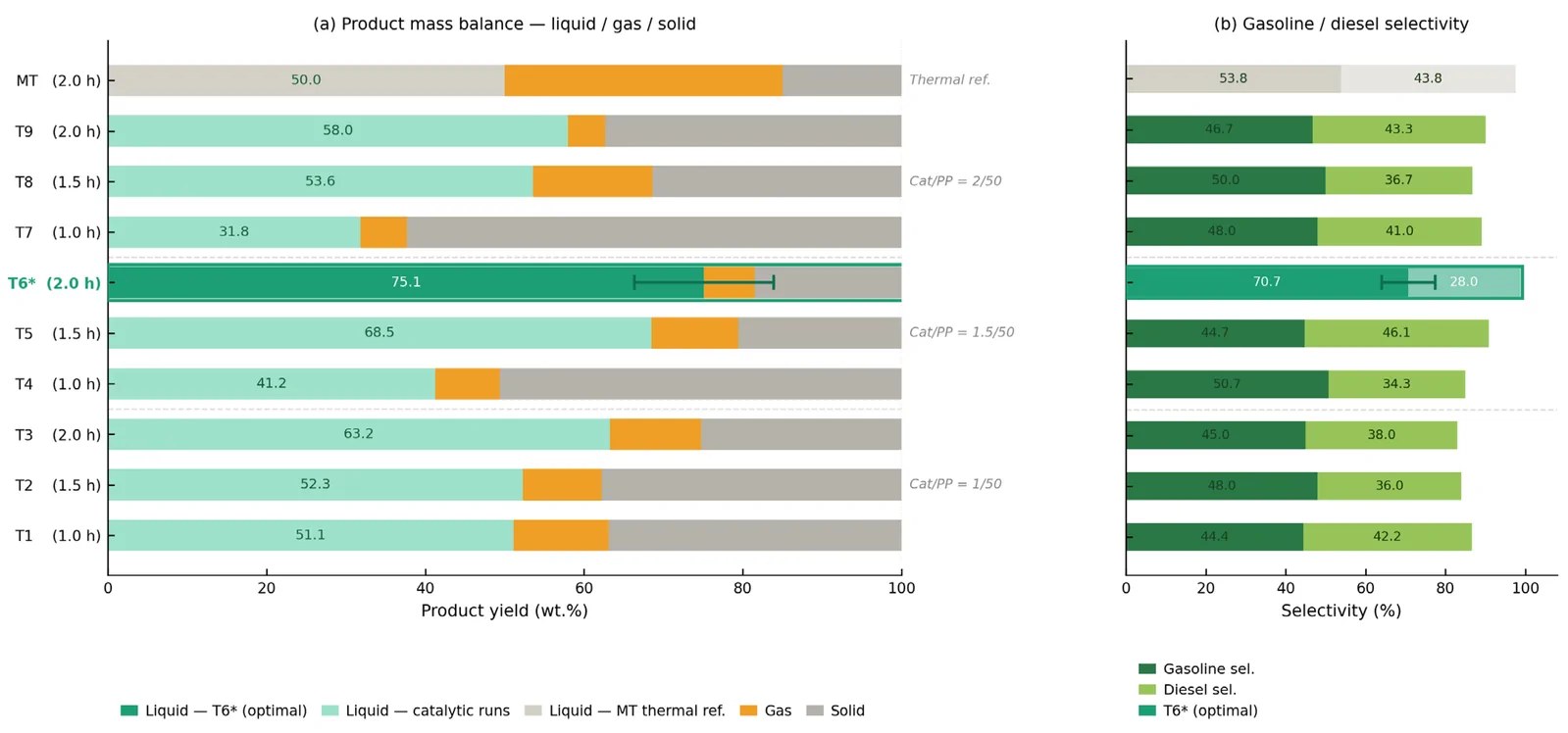
Product yield distribution and liquid fraction selectivity for pyrolysis runs T1–T9 and the thermal reference MT. (**a**) Mass balance (wt%); (**b**) gasoline and diesel selectivity (%). The asterisk (*) denotes the optimal treatment (run T6).

**Figure 5:**
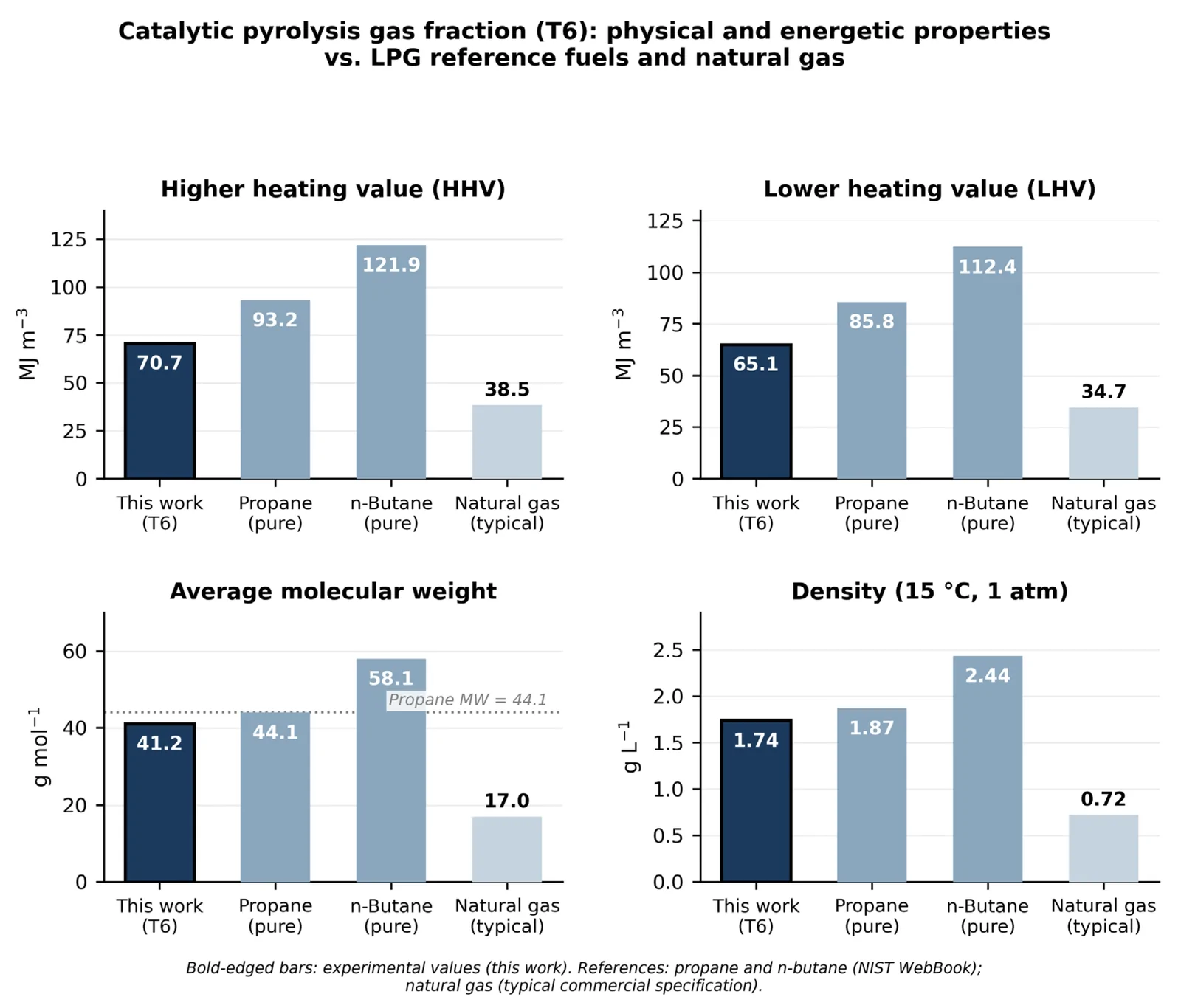
Physical and energetic properties of the catalytic gas fraction (T6) versus pure propane, pure n-butane, and typical natural gas.

**Figure 6:**
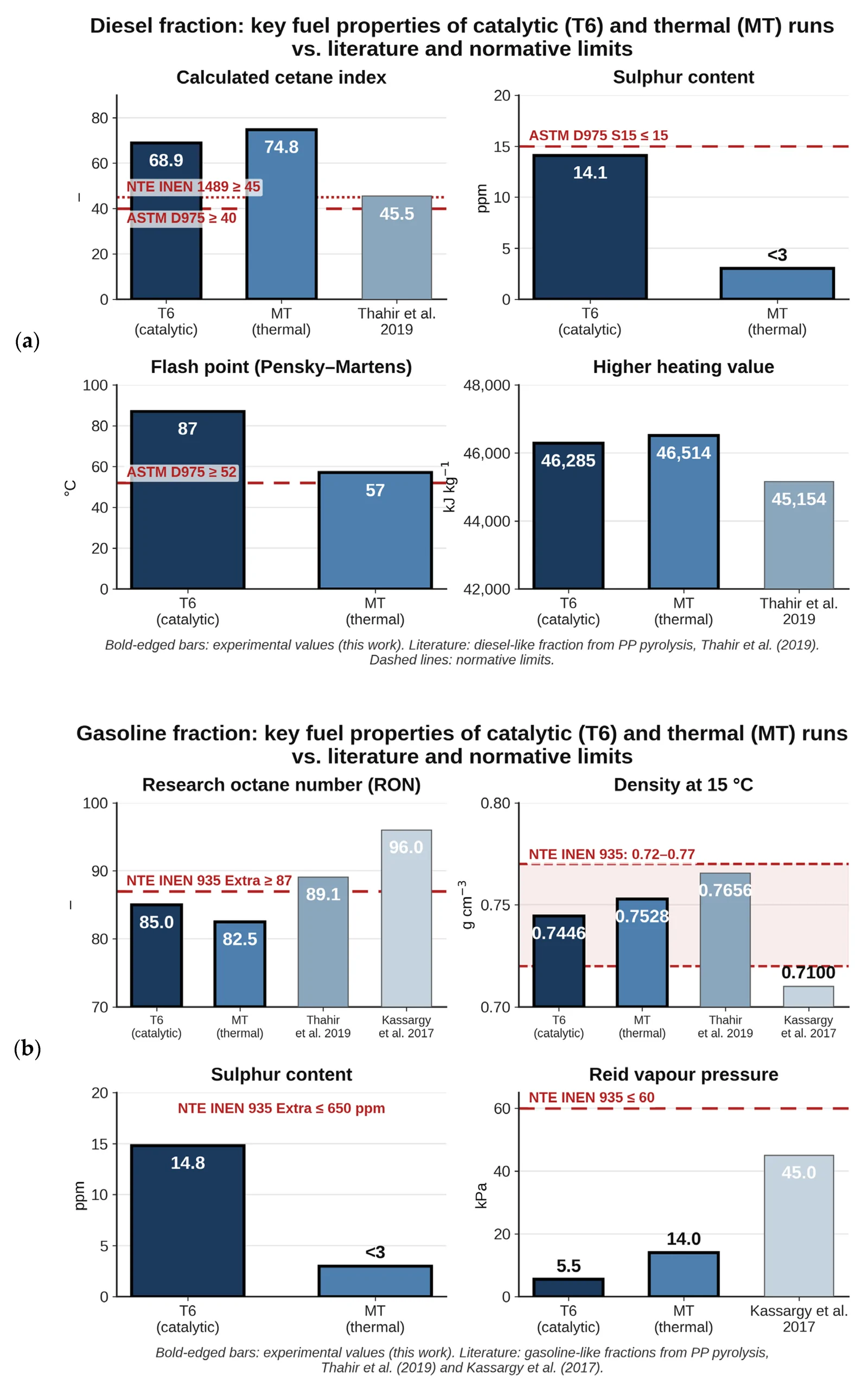
Key fuel properties of the catalytic (T6) and thermal (MT) (**a**) diesel and (**b**) gasoline fractions, compared with the literature and normative limits. Bold-edged bars are this work’s experimental values; literature data are from Thahir et al. (2019) [59] and Kassargy et al. (2017) [47]. Dashed lines indicate the ASTM D975, NTE INEN 1489, and NTE INEN 935 specification limits.

**Table 1:** Elemental compositions (wt%) and higher heating values of the three PP waste samples compared to virgin PP.

Parameter (wt%) ^a^	M1	M2	M3	Virgin PP [25]
Carbon	84.460	85.680	81.440	85.7
Hydrogen	15.100	14.037	16.905	14.3
Nitrogen	0.120	0.100	1.500	0.0
Sulphur	0.320	0.183	0.155	0.0
HHV (MJ kg^−1^) ^b^	44.14	45.27	44.63	44.0

^a^ Dry basis. Carbon, nitrogen, and sulphur were measured at the Laboratorio del Departamento de Petróleos, Energía y Contaminación (DPEC), Universidad Central del Ecuador, using an Elementar Vario Macro Cube analyser operated in combustion (Dumas) mode. Hydrogen was obtained by difference. The instrumental hydrogen readings (21.53, 21.94, and 20.63 wt% for M1, M2, and M3, respectively) were not retained because they yielded elemental sums of 106.43, 107.90, and 103.72 wt%, and because they exceeded the 14.37 wt% hydrogen content of a polyolefin backbone, which was identical for PP and PE. This indicates a systematic calibration bias of the hydrogen channel for this matrix; the values obtained by difference were consistent with the independently measured gross calorific values. Oxygen and ash were not determined and were taken as zero. ^b^ Gross calorific values determined by bomb calorimetry at the same laboratory (in-house procedure PNE/DPEC/P/M002, ref. ASTM D240), at ambient conditions of 543.1 mmHg and 19.5 °C, without correction for sulphur or moisture content.

**Table 2:** Characteristic TGA temperatures for the three PP waste samples at *β* = 30 °C min^−1^.

Sample	T_5_% (°C)	T_50_% (°C)	*T_max_* (°C)	T_95_% (°C)	Description
M1	387	456	473	500	Mixed PP/HDPE, chairs, containers
M2	368	439	450	481	Roofing covers (relatively pure PP)
M3	398	463	478	507	Commercial PP pellets with UV additives

**Table 3:** Physicochemical properties of fresh and regenerated FCC catalysts from an Ecuadorian refinery.

Property	Fresh FCC [43]	Regenerated FCC [43]	Fresh FCC [44] ^1^	Equilibrium FCC [44] ^2^	Regenerated FCC (This Study)
Bulk chemical composition (XRF [43,44]; EDS, this study)					
SiO_2_ (wt%)	34.65	39.04	56.2–65.3	47.0–47.6	45.62 ^3^
Al_2_O_3_ (wt%)	50.71	50.28	28.0–38.7	45.2–46.7	41.58 ^3^
Na_2_O (wt%)	0.10	0.12	0.19–0.42	0.53–0.67	0.19 ^3^
MgO (wt%)	0.35	0.14	–	–	3.69 ^3^
TiO_2_ (wt%)	0.22	0.19	0.21–0.66	0.27–0.38	0.90 ^3^
Bulk Si/Al molar ratio ^4^	0.58	0.66	1.2–2.0	0.86–0.89	0.93
Ni content (wt%)	–	2.05 ^5^	–	0.22–0.73	n.d. ^6^
V content (wt%)	–	0.06 ^5^	–	0.27–0.50	n.d. ^6^
Fe content (wt%)	0.44 ^7^	0.55 ^7^	0.27–0.82	0.36–0.90	4.84 ^3,7^
Rare-earth oxides (wt%)	1.18 ^8^	1.66 ^8^	2.39–5.46	2.16–3.65	3.14 ^3,8^
Textural properties (N_2_ physisorption)					
S_BET_ (m^2^ g^−1^)	158.13	76.78	–	–	134.1 ^9^
S_micro_/S_ext_ (m^2^ g^−1^)	63.87/94.25	38.23/38.55	–	–	–
Total pore volume (cm^3^ g^−1^)	0.147	0.098	–	–	0.067 ^9^
Micropore volume (cm^3^ g^−1^)	0.0328	0.0197	–	–	–
Microporosity (%)	40.39	49.79	–	–	–
Average pore width, 4V/S_BET_ (nm) ^10^	3.7	5.1	–	–	2.0
Acidity					
Brønsted + Lewis acid sites, Py-FTIR at 150 °C (μmol Py g^−1^) ^11^	210	69	–	–	–
Total acidity, NH_3_-TPD (μmol NH_3_ g^−1^) ^11^	–	–	–	–	66.1
Phase composition and morphology					
Zeolite Y content, XRD (%) ^12^	–	–	–	–	71.3
Zeolite Y unit-cell parameter a_0_ (Å) ^12^	–	–	–	–	24.256
La-exchanged faujasite, XRD (%)	–	–	–	–	2.2
Clay component ^13^	Kaolinite-1A	γ-Al_2_O_3_ + SiO_2_ (residual kaolin)	Kaolin (14–50 wt%)	Meta-kaolinite	Montmorillonite (26.5%)
Mean particle size (μm)	–	–	–	–	44.7

^1^ Commercial fresh FCC catalysts C1–C4 with 14–50 wt% kaolin (Fábrica Carioca de Catalisadores, Rio de Janeiro, Brazil) [53]. ^2^ Equilibrium catalysts (e-cats) sampled from two Brazilian refineries operating in full (RA) and partial (RB) combustion modes [53]. ^3^ Converted in this work to the oxide basis and normalised to 100 wt% from the semi-quantitative EDS elemental mass percentages measured for the regenerated catalyst at CENCINAT (as-measured: Si 21.01, Al 21.68, Fe 3.33, La 2.64, Mg 2.19, Ti 0.53, Na 0.14 wt%; oxide sum before normalisation: 98.5 wt%). ^4^ Calculated in this work from the SiO_2_ and Al_2_O_3_ contents reported by XRF in [52,53], and from the EDS Si and Al contents in this study; bulk values include the zeolite, clay filler, and alumina–silica matrix, and therefore differ from the framework Si/Al ratio of the zeolite phase (X: 1–1.5; Y: ≈2.5; USY: ≥6) [54]. ^5^ Reported as NiO and V_2_O_5_ in [52]. ^6^ n.d. = not detected by EDS. ^7^ Reported as Fe_2_O_3_ in [52]; total Fe in [53]; and converted from EDS Fe in this study. ^8^ Reported as La_2_O_3_ in [52]; total RE_2_O_3_ in [53]; and converted from EDS La in this study. ^9^ Single-point dynamic-flow N_2_ physisorption (Micromeritics AutoChem II 2920); values in [52] were obtained from multi-point N_2_ adsorption–desorption isotherms at 77 K (Micromeritics ASAP 2020, Micromeritics Instrument Corp., Norcross, GA, USA), so the comparison is indicative. ^10^ Average pore width estimated in this work as 4V_total_/S^BET^ from the values reported in each column. ^11^ Acidity values obtained by different techniques (pyridine-FTIR in [52]; NH_3_-TPD in this study) are not directly comparable and are shown for qualitative reference only. ^12^ Rietveld phase quantification (Cu Kα); the unit-cell parameter a_0_ = 24.256 Å is characteristic of strongly dealuminated (ultra stable) Y zeolite with the framework Si/Al ≥ 6 [54]. Refs. [52,53] do not report zeolite crystallinity for their samples. ^13^ Dominant clay/filler phase identified by XRD.

**Table 4:** GC-MS composition (area %) of pyrolysis liquids by the carbon number fraction from the best condition catalytic run T6 and thermal reference MT.

Fraction	Carbon Range	T6 (Area %)	MT Thermal (Area %)
Light aliphatics	C_4_–C_8_	18.22	10.88
C_9_ aliphatics	C_9_	23.28	25.35
Middle aliphatics	C_10_–C_12_	9.80	13.49
Diesel-range aliphatics	C_13_–C_19_	2.80	5.97
Wax fraction	C_20_+	0	16.43
**Identified aliphatics subtotal**	**—**	**54.10**	**72.12**
Aromatics	C_8_–C_9_	1.69	0.94
Oxygenates	—	5.55	6.47
Unidentified	—	38.66	20.46

**Table 5:** GC composition of gas fractions from thermal pyrolysis (MT, 2.0 h) and best condition catalytic run T6 (Cat/PP = 0.03 g g^−1^, 2.0 h, 450 °C, 0.45 bar abs)—determined by ASTM D 1945-03 (2014) and ASTM D 3588-98 (2017).

Compound	Thermal Pyrolysis % *w*/*w*	Catalytic Pyrolysis (T6) % *w*/*w*
Methane	2.33 ± 0.032	2.81 ± 0.079
CO_2_	10.06 ± 0.003	4.69 ± 0.004
Ethane	8.89 ± 0.003	8.96 ± 0.001
Propane	43.59 ± 0.095	46.25 ± 0.131
Water	0.01 ± 0.036	0.36 ± 0.052
Isobutane	0.22 ± 0.001	3.67 ± 0.000
n-Butane	6.85 ± 0.001	16.44 ± 0.001
Isopentane	24.49 ± 0.063	1.94 ± 0.001
n-Pentane	1.63 ± 0.001	14.47 ± 0.255
Hexane	1.92 ± 0.001	0.41 ± 0.001

**Table 6:** ASTM test methods, NTE INEN equivalent norms, and measured fuel properties of pyrolysis diesel and gasoline fractions from T6 (best condition run) compared to ASTM D975 Grade 2-D S15/NTE INEN 1489 (diesel) and NTE INEN 935 Extra (gasoline) specification limits.

Property	ASTM Method	NTE INEN Norm	Pyrolysis Diesel	ASTM D975 Grade 2-D S15	Pyrolysis Gasoline	NTE INEN 935 Extra
Sulphur (ppm)	D4294	1490	14.1	≤15	14.8	≤650
Density at 15 °C (g cm^−3^)	D4052	—	0.7813	0.82–0.85	0.7446	0.72–0.77
Kinematic viscosity 40 °C (mm^2^ s^−1^)	D445	810	2.565	1.9–4.1	—	—
Flash point (Pensky–Martens) (°C)	D93	1493	87	≥52	—	—
Calculated cetane index	D976	1495	68.92	≥40	—	—
Ash (wt%)	D482	1492	0.00056	≤0.01	—	—
Water and sediment (vol%)	D2709	1494	0.001	≤0.05	—	—
Conradson carbon residue (wt%)	D189/D4530	1491	0.044	≤0.10	—	—
Copper corrosion	D130	927	1A	≤3	1A	≤1
T_10_ distillation (°C)	D86	926	220.2	—	97.9	≤70
T_50_ distillation (°C)	D86	926	249.3	—	144.7	≤121
T_90_ distillation (°C)	D86	926	296.2	≤338	180.9	≤190
Final boiling point (°C)	D86	926	300.1	—	206.4	≤220
RON	D2699	2102	—	—	85	≥87
Reid vapour pressure (kPa)	D323	928	—	—	5.5	≤60
HHV (kJ kg^−1^)	D240	—	46,285	—	31,817	—

## Data Availability

The data supporting this study are available in the Supplementary Materials.

## Supplementary Materials

The following supporting information can be downloaded at: https://www.mdpi.com/article/10.3390/polym18212760/s1.

## Abbreviations

The following abbreviations are used in this manuscript:

PP	Polypropylene
iPP	Isotactic polypropylene
HDPE	High-density polyethylene
FCC	Fluid catalytic cracking
TGA	Thermogravimetric analysis
DTG	Derivative thermogravimetry
DSC	Differential scanning calorimetry
FTIR	Fourier transform infrared spectroscopy
GC–MS	Gas chromatography–mass spectrometry
SEM	Scanning electron microscopy
EDS	Energy-dispersive X-ray spectroscopy
XRD	X-ray diffraction
BET	Brunauer–Emmett–Teller surface area
NH_3_-TPD	Temperature-programmed desorption of ammonia
HHV	Higher heating value
LPG	Liquefied petroleum gas
RON	Research octane number
RSD	Relative standard deviation
