# Supplementary material for: Catalytic Pyrolysis of Post-Consumer Polypropylene over a Regenerated FCC Catalyst Under Moderate Vacuum: Gasoline- and Diesel-Range Fuels with a Low Aromatic Content
Source: Polymers (Basel). 2026 Nov 6;18(21):2760. doi: 10.3390/polym18212760 (PMC13765686; doi:10.3390/polym18212760)
Supplement: Supplementary file 1 [file polymers-18-02760-s001.zip › polymers-4559513-supplementary.pdf]

Supplementary Materials

Table S1. Ultimate analysis and experimental higher heating value (HHV) of the three post-consumer polypropylene feedstocks M1 (PP/HDPE), M2 (pure PP) and M3 (PP+UV).

Parameter	M1	M2	M3
Carbon (wt%)	84.460	85.680	81.440
Hydrogen (wt%)	15.100	14.037	16.905
Nitrogen (wt%)	0.120	0.100	1.500
Sulfur (wt%)	0.320	0.183	0.155
HHV (MJ kg^{-1})	44.14	45.27	44.63

Note: all determinations were carried out at the Laboratorio del Departamento de Petróleos, Energía y Contaminación (DPEC), Universidad Central del Ecuador. Carbon, nitrogen and sulfur were measured on an Elementar Vario Macro Cube analyser operated in combustion (Dumas) mode. Hydrogen was obtained by difference. The instrumental hydrogen readings (21.53, 21.94 and 20.63 wt.% for M1, M2 and M3, respectively) were not retained because they yielded elemental sums of 106.43, 107.90 and 103.72 wt.%, and because they exceed the 14.37 wt.% hydrogen content of a polyolefin backbone, which is identical for PP and PE. This indicates a systematic calibration bias of the hydrogen channel for this matrix; the values obtained by difference are consistent with the independently measured gross calorific values. Oxygen and ash were not determined and were taken as zero. HHV (gross calorific value) was determined by bomb calorimetry (in-house procedure PNE/DPEC/P/M002, ref. ASTM D240) at ambient conditions of 543.1 mmHg and 19.5 °C, without correction for sulfur or moisture content. The mean HHV of 44.68 ± 0.57 MJ kg^{-1} does not differ significantly from the reference value of 44 MJ kg^{-1} for virgin polypropylene (one-sample t-test: 95% confidence interval 43.28–46.08 MJ kg^{-1}, p = 0.172).

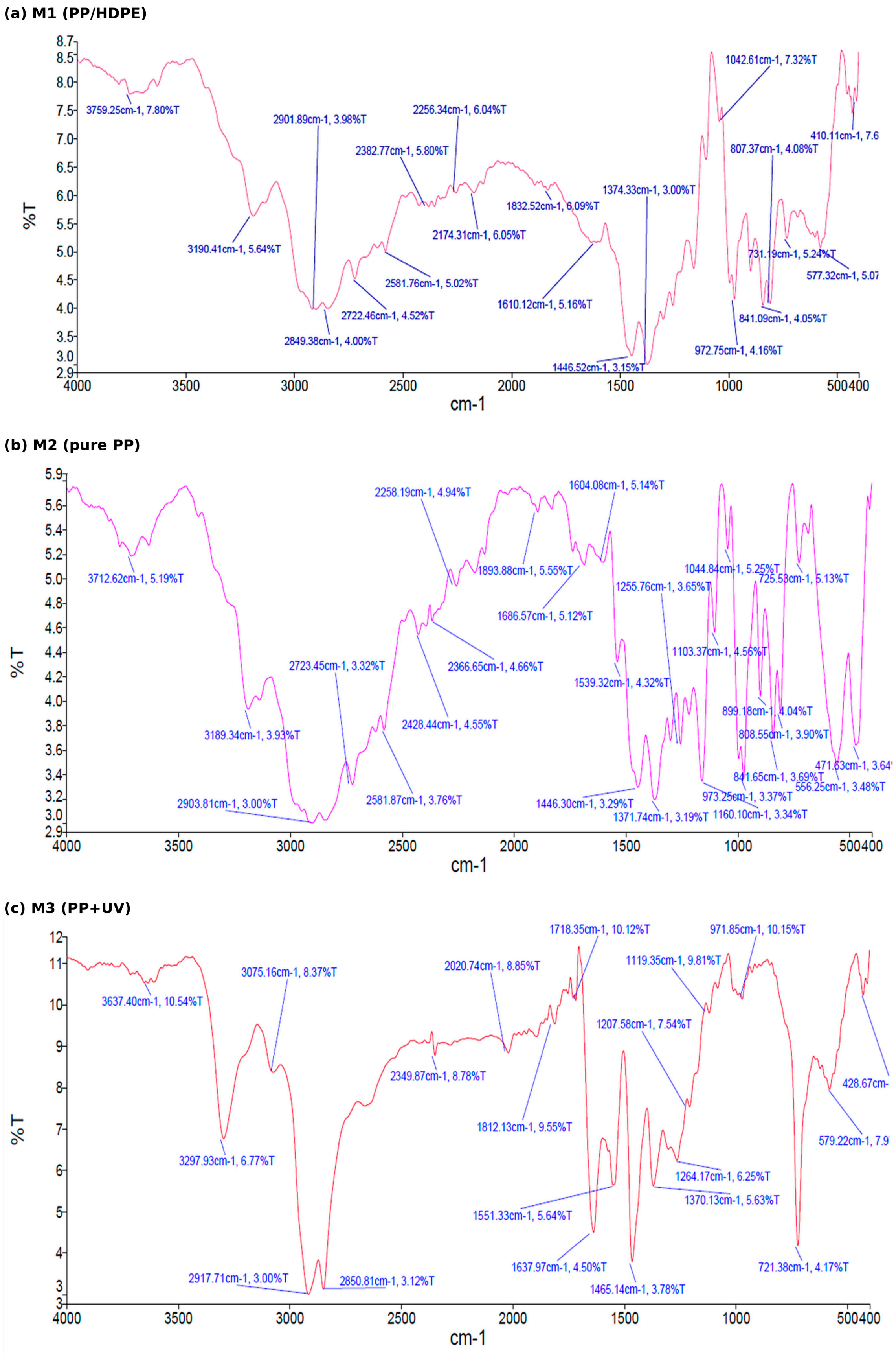

Figure S1. FTIR spectra of the three post-consumer polypropylene feedstocks: (a) M1 (PP/HDPE), (b) M2 (pure PP) and (c) M3 (PP+UV). Spectra recorded at the DPEC laboratory (Universidad Central del Ecuador). Peak labels correspond to the automatic annotations generated by the PerkinElmer Spectrum software; the representative bands used for structural assignment are compiled in Table S2.

Table S2. Representative FTIR absorption bands of the three post-consumer polypropylene feedstocks.

Bond / functional group	M1 (cm^{-1})	M2 (cm^{-1})	M3 (cm^{-1})
O–H···N (benzotriazole chelate)			3297.93
Aromatic C–H (UV stabiliser)			3075.16
$–CH_3$ / $–CH_2–$ aliphatic	2901.89; 2849.38	2903.81	2917.71; 2850.81
–SH alkyl mercaptans	2581.76	2581.87	
$–NH_3$ amines	2382.77	2366.65	2349.87
–N=C=O isocyanates	2256.34	2258.19	
C≡N isonitriles	2174.31		
C=O carbonyl		1686.57	
C≡C; $C_6H_5–$	1610.12	1604.08	
Triazole ring C=N (UV stabiliser)			1551.33
$–CH_3$ aliphatic	1446.52	1446.30	1465.14
Isopropyl	1374.33	1371.74	1370.13
C–N stretch (UV stabiliser)			1264.17
iPP		1255.76; 1103.37; 899.18	1119.35
iPP; C=O			1207.58
iPP	1042.61	1044.84	
iPP; C=C	972.75	973.25	971.85
iPP	841.09; 807.37	841.65; 808.55	
$–CH_2$ long methylene chains	731.19	725.53	721.38
–Cl–C=O chlorinated acids			428.67

Note: the bands in the 1250–835 cm^{-1} region confirm an isotactic polypropylene (iPP) backbone in the three feedstocks, with M2 showing the largest number of iPP-characteristic bands and no evidence of polyethylene. The polyethylene fraction of M1 is evidenced by the CH_2 stretching bands at 2901.89 and 2849.38 cm^{-1} together with the CH_2 rocking band shifted to 731.19 cm^{-1}, relative to the single rocking band at 725.53 cm^{-1} in M2. In M3 the bands at 3297.93 (intramolecular O–H···N hydrogen bond of the benzotriazole chelate), 3075.16 (aromatic C–H), 1551.33 (triazole ring C=N) and 1264.17 cm^{-1} (C–N stretch) jointly identify the benzotriazole UV stabiliser 2,4-di-tert-butyl-6-(5-chloro-2H-benzotriazol-2-yl)phenol. The weak, sharp features observed between 3600 and 3760 cm^{-1} in all three spectra (Figure S1) are attributed to uncompensated atmospheric water vapour and were therefore excluded from the structural assignment. The chlorinated-acid band at 428.67 cm^{-1} is exclusive to M3. The bands recorded near 3190 cm^{-1} in M1 and M2 are not diagnostic of polyethylene and were left unassigned. Blank cells indicate that the band was not detected in that sample.

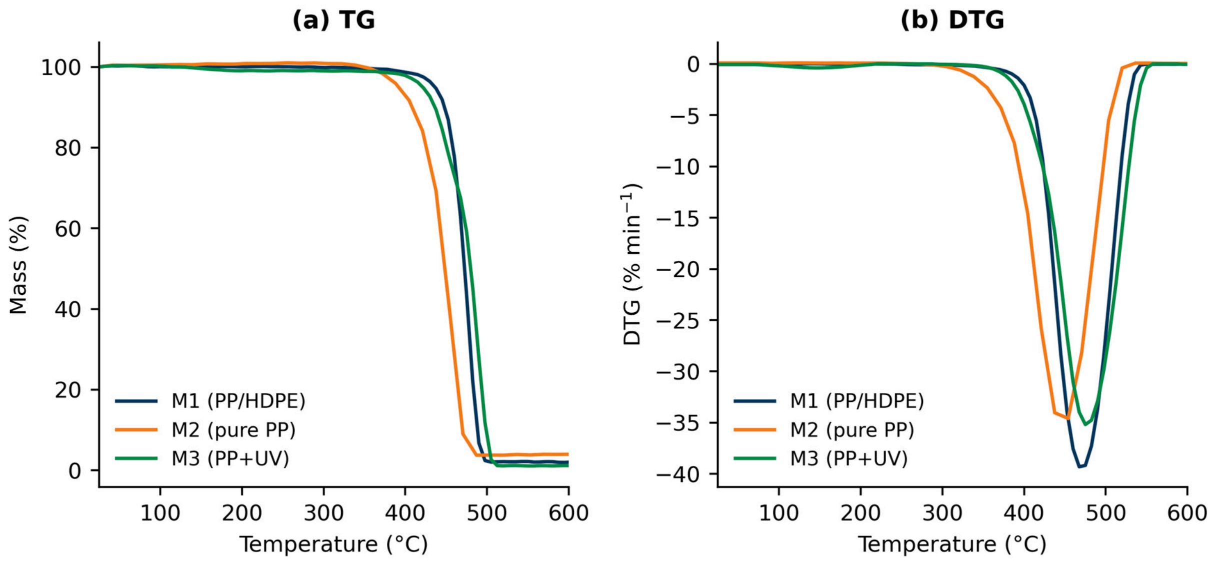

Figure S2. TG (a) and DTG (b) curves of the three post-consumer polypropylene samples M1 (PP/HDPE), M2 (pure PP) and M3 (PP+UV) under N_2 atmosphere at β = 30 °C min^{-1}.

Table S3. Thermogravimetric parameters of the three post-consumer polypropylene feedstocks (β = 30 °C min^{-1}, N_2).

Parameter	M1	M2	M3
T_{max} (°C)	473	450	478
Maximum conversion (wt%)	98.05	96.25	99.11
Shift in Tmax relative to neat PP (°C)	+48	+25	+53

Note: T_{max} = temperature of the single DTG peak. Mean T_{max} of the three feedstocks = 467 °C; approximate residence time required to reach complete devolatilisation = 16.25 min. An Anderson–Darling normality test on T_{max} gives p = 0.217 (α = 0.05), consistent with a common polypropylene backbone across the three feedstocks. The shift is referred to the T_{max} of 425 °C reported for neat polypropylene (Buekens, 2006), against 475 °C for neat HDPE; the three feedstocks therefore fall between these two reference values. No DTG peak was observed below 100 °C, indicating the absence of free moisture.

Table S4. Coats–Redfern first-order kinetic parameters (T^2 integral form) from the linear regression of ln[−ln(1−α)/T²] vs 1/T for feedstocks M1–M3 (β = 30 °C min^{-1}, N_2).

Sample	Linear regression	R^2	E_a (kJ mol^{-1})	A (s^{-1})
M1	$\ln[-\ln(1-\alpha)/T^2] = -42327/T + 43.267$	0.9963	351.91	1.35×10^{23}
M2	$\ln[-\ln(1-\alpha)/T^2] = -21445/T + 16.346$	0.9893	178.29	1.44×10^{11}
M3	$\ln[-\ln(1-\alpha)/T^2] = -25165/T + 19.918$	0.9974	209.22	5.98×10^{12}

Note: T in K; first-order reaction; single non-isothermal experiment at β = 30 °C min^{-1}, N_2. Slope = $-E_a/R$, intercept = $\ln(AR/\beta E_a)$.

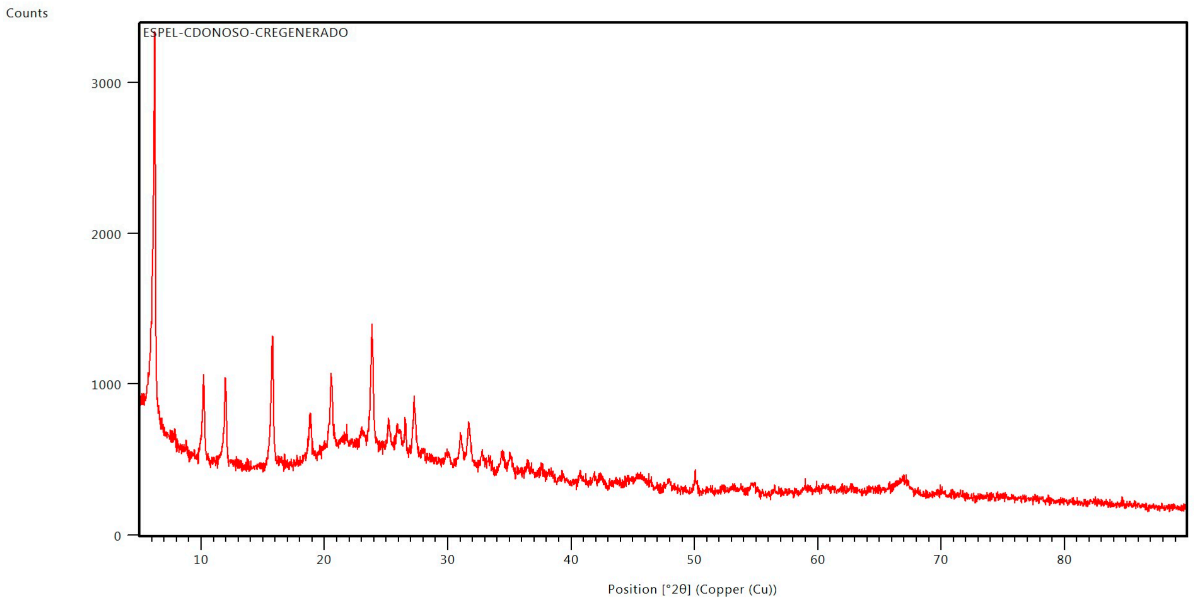

Figure S3. X-ray diffraction pattern of the regenerated FCC catalyst (Cu Kα). Rietveld-based phase quantification: zeolite Y 71.3% (a_0 = 24.2560 Å), La-exchanged dehydrated faujasite 2.2% (a_0 = 24.7300 Å) and montmorillonite 26.5% (a = 5.18 Å, b = 8.98 Å, c = 15.00 Å).

Table S5. Semi-quantitative EDS elemental composition of the regenerated FCC catalyst (TESCAN MIRA3, CENCINAT-ESPE) and conversion to the normalized oxide basis.

Element	As measured (wt%)	Oxide	Normalized oxide basis (wt%)
Si	21.01	SiO_2	45.62
Al	21.68	Al_2O_3	41.58
Fe	3.33	Fe_2O_3	4.84
La	2.64	La_2O_3	3.14
Mg	2.19	MgO	3.69
Ti	0.53	TiO_2	0.90
Na	0.14	Na_2O	0.19
Zn	0.03	ZnO	0.04

Note: carbon and sulfur were excluded from quantification. Oxide values were normalized to 100 wt% (sum before normalization: 98.5 wt%).

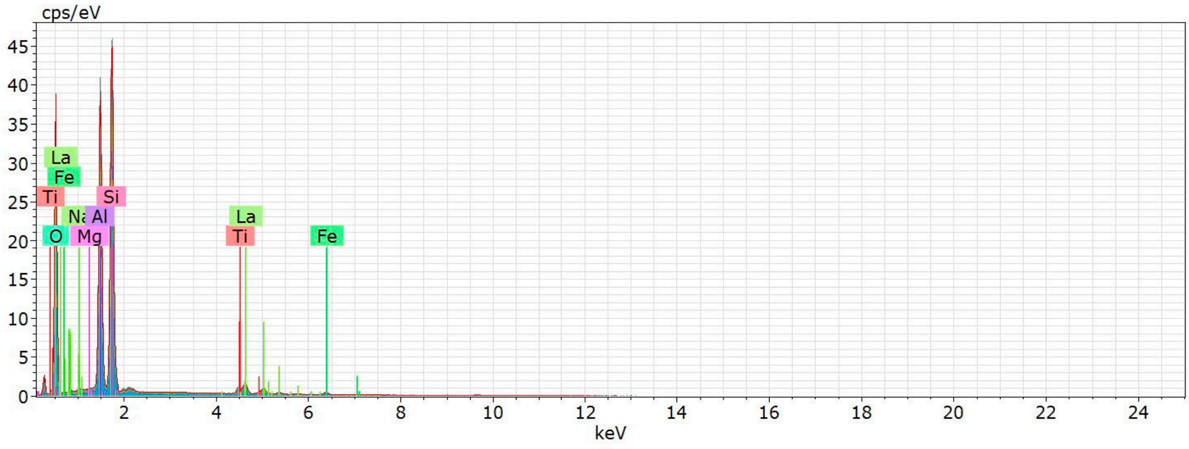

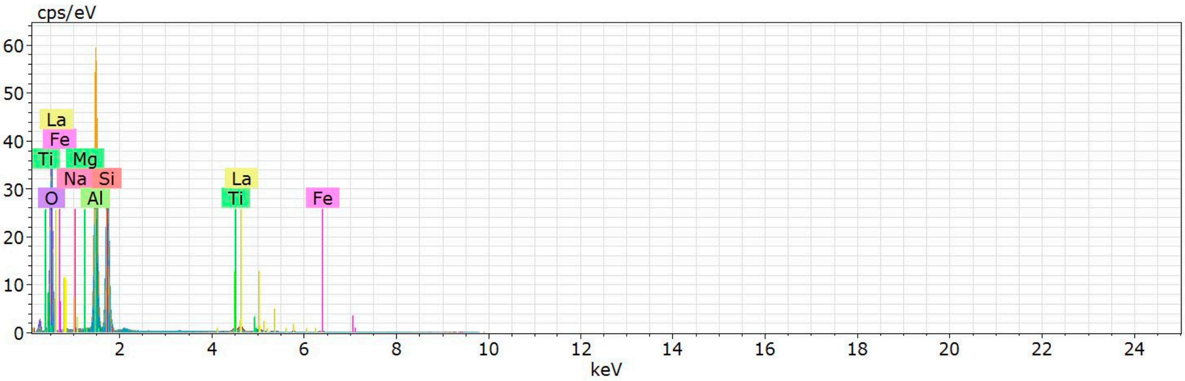

Figure S4. EDS spectra of the FCC catalyst samples (TESCAN MIRA3, CENCINAT-ESPE; C and S excluded from quantification): (a) regenerated (calcined) catalyst; (b) spent catalyst.

Table S6. GC–MS peak assignments of the T6 (catalytic, Cat/PP = 1.5/50 g g⁻¹, 2 h) liquid fraction. Blank compound entries denote peaks without a reliable NIST library match (unidentified).

#	Area %	Norm. %	Compound	Formula
1	1.259	15.46	isobutylene	C_4H_8
2	4.905	60.24	2-methylpentanal	$C_6H_{12}O$
3	1.347	16.54	2-methyl-2-butene	C_5H_{10}
4	1.426	17.52	2-pentene	C_5H_{10}
5	2.152	26.43	2-methyl-1-pentene	C_6H_{12}
6	2.163	26.56	2,3-dimethyl-1-butene	C_6H_{12}
7	0.660	8.10	2,4-dimethyl-1-pentene	C_7H_{14}
8	1.135	13.94	3,4-dimethyl-2-pentene (Z)	C_7H_{14}
9	0.586	7.20	heptanal	$C_7H_{14}O$
10	1.584	19.45	2,4-dimethyl-2,3-pentadiene	C_7H_{12}
11	0.584	7.18	2,3-dimethyl-2-hexene	C_8H_{16}
12	0.447	5.49	3,5-dimethylcyclopentene	C_7H_{12}
13	0.503	6.18	4-methyleneheptane	C_8H_{16}
14	0.563	6.92	2,5-dimethyl-2-hexene	C_8H_{16}
15	2.264	27.80	4-methylheptane	C_8H_{18}
16	0.466	5.72	4-methyl-2-heptene	C_8H_{16}
17	0.515	6.32	4-methyl-3-heptene	C_8H_{16}
18	0.959	11.78	3,5-dimethyl-1-cyclohexene	C_8H_{14}
19	0.660	8.11	1,1,3,4-tetramethylcyclopentane (cis)	C_9H_{18}
20	1.047	12.85	2-cyclohexylpropane	C_9H_{18}
21	6.253	76.79	propylcyclohexane	C_9H_{18}
22	0.625	7.67	1,3,5-trimethylcyclohexane	C_9H_{18}
23	8.143	100	2,4-dimethyl-1-heptene	C_9H_{18}
24	0.747	9.17	2,3-dimethyl-3-heptene (Z)	C_9H_{18}
25	0.679	8.33	2,3-dimethyl-3-heptene (Z)	C_9H_{18}
26	0.402	4.94	1,1,3,4-tetramethylcyclopentane (cis)	C_9H_{18}
27	1.229	15.09	1,2,4-trimethylcyclohexane	C_9H_{18}
28	0.447	5.50	3,3-dimethyl-1,6-heptadiene	C_9H_{16}
29	1.184	14.54	3,3,5-trimethylcyclohexene	C_9H_{16}
30	1.614	19.82	4,4,5-trimethyl-2-hexene	C_9H_{18}
31	0.527	6.47	Unidentified	C_9H_{18}
32	1.456	17.88	1,1,2-trimethyl-3-(2-methylpropyl)cyclopropane	$C_{10}H_{20}$
33	0.453	5.57	1,4,6,6-tetramethylcyclohexene	$C_{10}H_{18}$
34	0.547	6.72	(1S,2S,3R)-2-isopropenyl-1,3-dimethylcyclopentane	$C_{10}H_{18}$
35	0.440	5.41	2,6-dimethyl-2-octene	$C_{10}H_{20}$
36	0.763	9.38	2,6-dimethyl-2-octene	$C_{10}H_{20}$
37	0.434	5.34	2,6-dimethyl-2-octene	$C_{10}H_{20}$
38	0.510	6.26	1,2,4-trimethylbenzene	C_9H_{12}
39	1.159	14.23	3,3-dimethyloctane	$C_{10}H_{20}$

#	Area %	Norm. %	Compound	Formula
40	1.076	13.22	4-methyldecane	$C_{11}H_{24}$
41	0.417	5.12	1-butyl-2-propylcyclopentane	$C_{11}H_{22}$
42	1.255	15.42	2,4-dimethyl-2-decene	$C_{12}H_{24}$
43	1.167	14.33	2,4-dimethyl-2-decene	$C_{12}H_{24}$
44	1.477	18.14	Unidentified	–
45	1.323	16.25	Unidentified	–
46	0.603	7.40	Unidentified	–
47	0.413	5.07	Unidentified	–
48	0.423	5.19	1-butyl-2,4,6-trimethylcyclohexane	$C_{13}H_{26}$
49	0.484	5.94	Unidentified	–
50	0.432	5.30	1-(1,1-dimethylethyl)-2-(1,2,2-trimethylpropylidene)cyclopropane (Z)	$C_{13}H_{24}$
51	0.699	8.58	Unidentified	–
52	0.782	9.60	Unidentified	–
53	2.066	25.38	Unidentified	–
54	0.714	8.77	Unidentified	–
55	0.531	6.53	1-methyl-3-propylcyclooctane	$C_{12}H_{24}$
56	1.378	16.92	Unidentified	–
57	1.982	24.34	Unidentified	–
58	2.688	33.01	Unidentified	–
59	1.742	21.39	Unidentified	–
60	1.913	23.50	1-nonadecene	$C_{19}H_{38}$

Table S7. GC–MS peak assignments of the MT (thermal, 2.5 h) liquid fraction. Blank compound entries denote peaks without a reliable NIST library match (unidentified).

#	Area %	Norm. %	Compound	Formula
1	0.359	1.69	isobutylene	C_4H_8
2	3.763	17.68	3-propoxy-1-propene	$C_6H_{12}O$
3	0.675	3.17	2-methyl-1-butene	C_5H_{10}
4	2.990	14.05	2-methyl-1-pentene	C_6H_{12}
5	0.425	2.00	2,3-dimethyl-2-butene	C_6H_{12}
6	0.480	2.26	2-methyl-1,3-pentadiene (E)	C_6H_{10}
7	0.339	1.59	2,4-dimethyl-1-pentene	C_7H_{14}
8	0.666	3.13	2,4-dimethyl-1,4-pentadiene	C_6H_{12}
9	0.399	1.88	2,4-dimethyl-1,3-pentadiene	C_7H_{12}
10	0.570	2.68	2-cyclopropyl-3-methylbutane	C_8H_{16}
11	3.531	16.59	2,3-dimethylhexane	C_8H_{18}
12	0.292	1.37	2-methyl-1-heptene	C_8H_{16}
13	0.399	1.87	1,2,3-trimethylcyclopentene	C_8H_{14}
14	1.017	4.78	1,1,3,4-tetramethylcyclopentane (cis)	C_9H_{18}
15	0.323	1.52	2,3,4-trimethylhexane	C_9H_{20}
16	21.286	100	2,4-dimethyl-1-heptene	C_9H_{18}
17	3.052	14.34	1,3,5-trimethylcyclohexane	C_9H_{18}
18	0.331	1.55	ethylbenzene	C_8H_{10}
19	0.656	3.08	3,3,5-trimethylcyclohexene	C_9H_{16}
20	0.523	2.46	3,3,5-trimethylcyclohexene	C_9H_{16}
21	5.578	26.20	p-xylene-2-sulfonic acid	$C_8H_{10}O_3S$
22	0.381	1.79	5,7-dimethyl-1,6-octadiene	$C_{10}H_{18}$
23	0.487	2.29	2,7-dimethyl-1,6-octadiene	$C_{10}H_{18}$
24	0.260	1.22	2,6-dimethyl-2-octene	$C_{10}H_{20}$
25	0.384	1.80	4-methylnonane	$C_{10}H_{20}$
26	0.610	2.87	α-methylstyrene	C_9H_{10}
27	0.618	2.90	Unidentified	$C_8H_{18}O$
28	0.242	1.14	Unidentified	–
29	1.650	7.75	3,3,5-trimethylheptane	$C_{10}H_{22}$
30	1.731	8.13	3,3-dimethyloctane	$C_{10}H_{22}$
31	0.317	1.49	nonylcyclopropane	$C_{12}H_{24}$
32	0.290	1.36	1-ethyl-2,2,6-trimethylcyclohexane	$C_{11}H_{22}$
33	0.594	2.79	Unidentified	$C_{22}H_{40}O_4$
34	0.424	1.99	Unidentified	–
35	0.331	1.55	Unidentified	$C_{10}H_{20}O$
36	5.377	25.26	7-methyl-1-undecene	$C_{12}H_{24}$
37	4.225	19.85	(3E)-3-octadecene	$C_{18}H_{36}$
38	0.378	1.77	7-tetradecene	$C_{14}H_{28}$
39	0.265	1.24	2,6,11-trimethyldodecane	$C_{15}H_{32}$

#	Area %	Norm. %	Compound	Formula
40	0.243	1.14	3-(cyclopentylmethyl)-3,7-dimethylocta-1,6-diene	$C_{16}H_{28}$
41	0.325	1.53	1,3-diisopropylcyclohexane	$C_{12}H_{24}$
42	0.694	3.26	Unidentified	–
43	0.579	2.72	1-methyl-3-propylcyclooctane	$C_{12}H_{24}$
44	1.503	7.06	(2,4,6-trimethylcyclohexyl)methanol	$C_{10}H_{20}O$
45	0.758	3.56	(2,4,6-trimethylcyclohexyl)methanol	$C_{10}H_{20}O$
46	0.793	3.73	1-isopropyl-1,4,5-trimethylcyclohexane	$C_{12}H_{24}$
47	0.394	1.85	1,3-diisopropylcyclohexane	$C_{12}H_{24}$
48	0.257	1.21	(2,4,6-trimethylcyclohexyl)methanol	$C_{10}H_{20}O$
49	0.275	1.29	cyclododecane	$C_{12}H_{24}$
50	0.543	2.55	1-isopropyl-1,4,5-trimethylcyclohexane	$C_{12}H_{24}$
51	0.275	1.29	Unidentified	–
52	0.996	4.68	nonadecane	$C_{19}H_{40}$
53	0.598	2.81	Unidentified	–
54	0.579	2.72	2,6,10,15-tetramethylheptadecane	$C_{21}H_{44}$
55	0.270	1.27	1-tetracosene	$C_{24}H_{48}$
56	0.288	1.36	1-tetracosene	$C_{24}H_{48}$
57	0.331	1.56	(2,4,6-trimethylcyclohexyl)methanol	$C_{10}H_{20}O$
58	0.773	3.63	1-tetracosene	$C_{24}H_{48}$
59	9.573	44.97	cyclotetracosane	$C_{24}H_{48}$
60	5.312	24.96	cyclotetracosane	$C_{24}H_{48}$

Note to Tables S6–S7: compound names standardized to English nomenclature from the original GC–MS report; Norm. % = peak area normalized to the base peak (2,4-dimethyl-1-heptene = 100%).

NH_3 temperature-programmed desorption (total acidity). The total acidity of the regenerated FCC catalyst was determined by NH_3-TPD on a Micromeritics AutoChem II 2920 analyser with thermal conductivity detection (TCD), using 0.200 g of sample. Following ammonia saturation and helium purging to remove physisorbed NH_3 at 120 °C, the temperature was ramped at 5 °C min^{-1} up to 700 °C under a helium flow of 25.07 cm^3 STP min^{-1}, and the desorption profile was processed with the MicroActive software. The TCD response was calibrated by injecting known ammonia volumes (0.1–1 mL loop). The total acid-site density was obtained by integrating the NH_3 desorption peak between 120 and 260 °C with a zero baseline, yielding 66.1 μmol NH_3 g^{-1} (Table S8). The desorption profile (Figure S5) shows a single maximum at *Tmax* ≈ 230 °C, characteristic of acid sites of predominantly weak-to-moderate strength; the monotonically rising signal above 260 °C corresponds to TCD baseline drift and was excluded from the quantification.

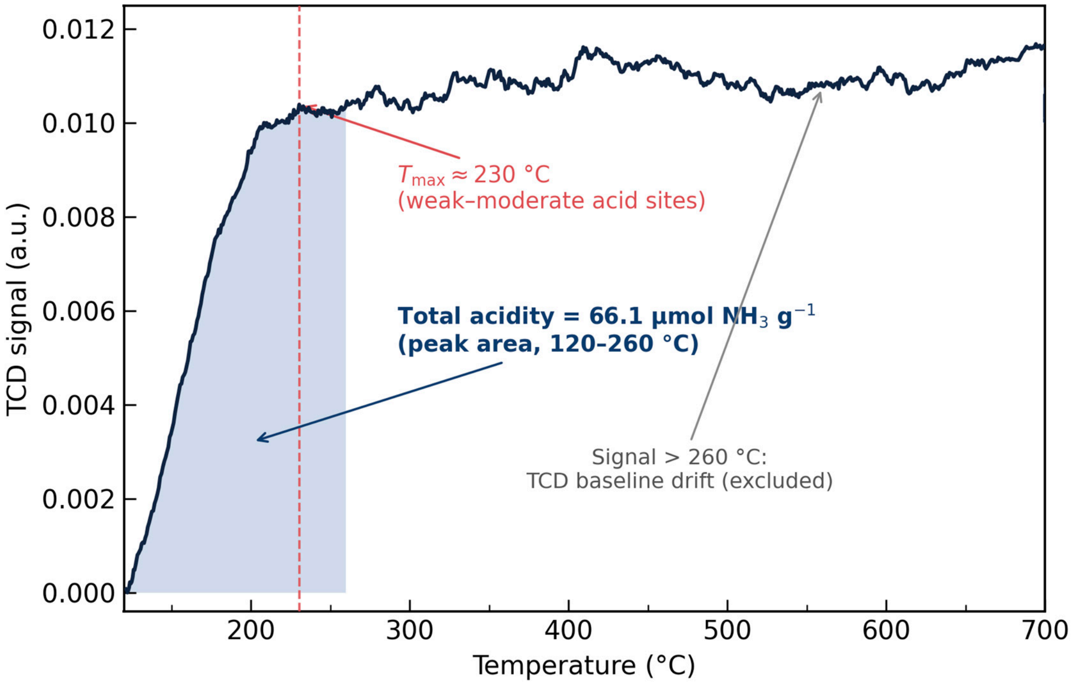

Figure S5. NH_3-TPD profile of the regenerated FCC catalyst (TCD signal vs temperature, 5 °C min^{-1}). A single desorption maximum at *Tmax* ≈ 230 °C indicates predominantly weak-to-moderate acid sites. Total acidity (66.1 μmol NH_3 g^{-1}) was obtained by integrating the desorption peak over 120–260 °C (shaded); the elevated signal above 260 °C corresponds to TCD baseline drift and was excluded from quantification.

Table S8. NH_3-TPD operating conditions and derived total acidity (regenerated FCC catalyst).

**Parameter**	**Value**	**Unit**
Instrument / detector	Micromeritics AutoChem II 2920 / TCD	—
Sample mass	0.200	g
Carrier gas, measured flow	He, 25.07	cm^3 STP min^{-1}
Heating rate	5	°C min^{-1}
Temperature range	120–700	°C
NH_3 calibration loop	0.1–1	mL
Integration window (acid quantification)	120–260	°C
Total acidity	66.1	μmol NH_3 g^{-1}

Estimated elemental composition of the pyrolysis oils. Direct CHNS/O analysis of the liquid products was not performed; instead, the elemental composition of the oils was estimated from the GC–MS speciation (Tables S6–S7) following the peak-area/brute-formula approach of Nardella et al. [56]. Each identified compound was weighted by its chromatographic area and the overall C, H and O contents and the H/C and O/C atomic ratios were computed from the molecular formulas; unidentified peaks and one implausible sulfonic-acid library assignment were excluded (identified-area coverage: 79 % for the catalytic oil, 92 % for the thermal oil). Both oils give H/C ≈ 2.0, matching the polypropylene backbone, and O/C ≤ 0.011, i.e. a predominantly hydrocarbon oil with only minor oxygenates (Table S9). This is the expected signature of pure-PP pyrolysis and contrasts with the oxygen-rich oils reported for biomass–plastic co-pyrolysis, for which the same estimation method was originally developed. Sulfur is better represented by the direct measurement on the oil (14.1–14.8 ppm); the GC–MS-based estimate is not reliable for trace heteroatoms.

Table S9. Estimated elemental composition of the pyrolysis oils from GC–MS speciation (area-weighted brute formulas, after Nardella et al. [56]), compared with the theoretical composition of polypropylene.

Oil	C (wt%)	H (wt%)	O (wt%)	H/C	O/C
T6 (catalytic)	84.7	14.1	1.2	1.98	0.011
MT (thermal)	84.7	14.2	1.1	2.00	0.009
PP feedstock (theoretical)	85.6	14.4	0	2.00	0

[56] Nardella, F.; Bellavia, S.; Mattonai, M.; Ribechini, E. Co-pyrolysis of biomass and plastic: Synergistic effects and estimation of elemental composition of pyrolysis oil by analytical pyrolysis–gas chromatography/mass spectrometry. Bioresour. Technol. 2022, 354, 127170. https://doi.org/10.1016/j.biortech.2022.127170.

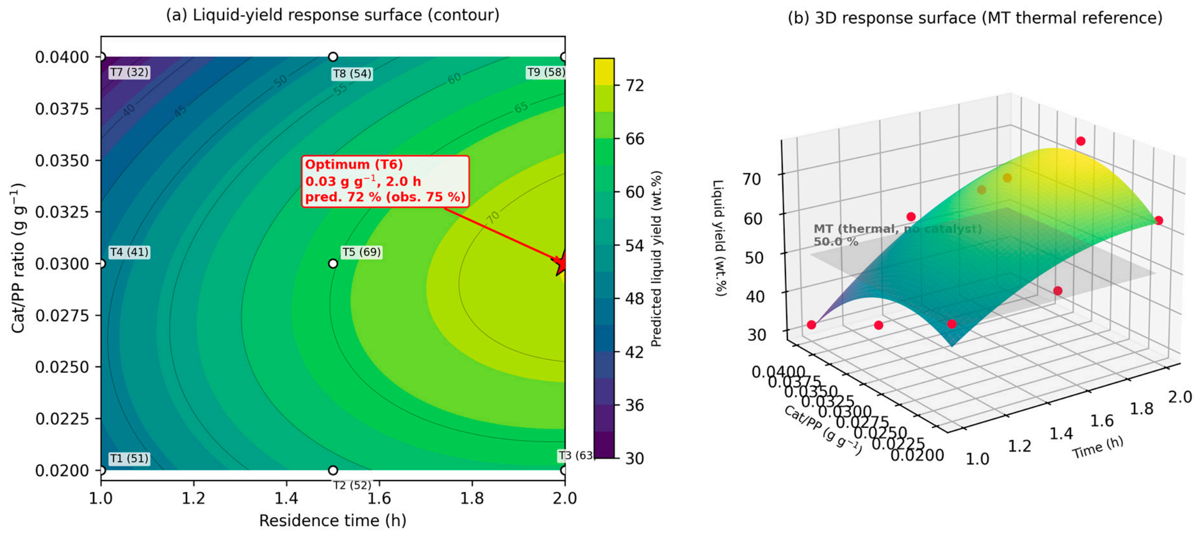

Figure S6. Response surface of the liquid-fraction yield as a function of the catalyst/PP ratio and residence time (450 °C, 0.45 bar). (a) Contour plot with treatments T1–T9 and the best condition within the domain (T6); (b) 3D surface with the MT thermal reference plane (50.0 %). Quadratic model fitted to the 27 observations (triplicates).
